# Birch Pollen Induces Toll-Like Receptor 4-Dependent Dendritic Cell Activation Favoring T Cell Responses

**DOI:** 10.3389/falgy.2021.680937

**Published:** 2021-08-12

**Authors:** Lisa Pointner, Amin Kraiem, Michael Thaler, Fabian Richter, Mario Wenger, Athanasios Bethanis, Markus Klotz, Claudia Traidl-Hoffmann, Stefanie Gilles, Lorenz Aglas

**Affiliations:** ^1^Department of Biosciences, University of Salzburg, Salzburg, Austria; ^2^Chair of Environmental Medicine, Faculty of Medicine, University of Augsburg, Augsburg, Germany; ^3^Institute of Environmental Medicine, Helmholtz Zentrum München, Neuherberg, Germany; ^4^Christine Kühne 96 Center for Allergy Research and Education (CK-CARE), Davos, Switzerland

**Keywords:** allergic sensitization, T cell response, dendritic cells, innate immunity, TLR4 signaling, birch pollen, lipopolysaccharide, toll-like receptor 4

## Abstract

Seasonal exposure to birch pollen (BP) is a major cause of pollinosis. The specific role of Toll-like receptor 4 (TLR4) in BP-induced allergic inflammation and the identification of key factors in birch pollen extracts (BPE) initiating this process remain to be explored. This study aimed to examine (i) the importance of TLR4 for dendritic cell (DC) activation by BPE, (ii) the extent of the contribution of BPE-derived lipopolysaccharide (LPS) and other potential TLR4 adjuvant(s) in BPE, and (iii) the relevance of the TLR4-dependent activation of BPE-stimulated DCs in the initiation of an adaptive immune response. *In vitro*, activation of murine bone marrow-derived DCs (BMDCs) and human monocyte-derived DCs by BPE or the equivalent LPS (nLPS) was analyzed by flow cytometry. Polymyxin B (PMB), a TLR4 antagonist and TLR4-deficient BMDCs were used to investigate the TLR4 signaling in DC activation. The immunostimulatory activity of BPE was compared to protein-/lipid-depleted BPE-fractions. In co-cultures of BPE-pulsed BMDCs and Bet v 1-specific hybridoma T cells, the influence of the TLR4-dependent DC activation on T cell activation was analyzed. *In vivo* immunization of IL-4 reporter mice was conducted to study BPE-induced Th2 polarization upon PMB pre-treatment. Murine and human DC activation induced by either BPE or nLPS was inhibited by the TLR4 antagonist or by PMB, and abrogated in TLR4-deficient BMDCs compared to wild-type BMDCs. The lipid-free but not the protein-free fraction showed a reduced capacity to activate the TLR4 signaling and murine DCs. In human DCs, nLPS only partially reproduced the BPE-induced activation intensity. BPE-primed BMDCs efficiently stimulated T cell activation, which was repressed by the TLR4 antagonist or PMB, and the addition of nLPS to Bet v 1 did not reproduce the effect of BPE. *In vivo*, immunization with BPE induced a significant Th2 polarization, whereas administration of BPE pre-incubated with PMB showed a decreased tendency. These findings suggest that TLR4 is a major pathway by which BPE triggers DC activation that is involved in the initiation of adaptive immune responses. Further characterization of these BP-derived TLR4 adjuvants could provide new candidates for therapeutic strategies targeting specific mechanisms in BP-induced allergic inflammation.

## Introduction

Birch pollen (BP) is one of the most dominant allergenic sources in the Northern Hemisphere, including Europe and North America, and accounts for high sensitization rates among tree-pollen allergic individuals, with Bet v 1 being the major allergen (1, 2). Seasonal exposure to BP can cause allergic sensitization in genetically predisposed individuals, characterized by the differentiation of T helper type 2 (Th2) lymphocytes and the production of allergen-specific immunoglobulin (Ig)E antibodies, further driving disease progression and resulting in allergic symptoms (3). However, the initial molecular mechanisms triggering the onset of allergic sensitization and the key factors in BP promoting this process remain unclear.

To address this matter, the studies of early immunological events occurring when BP encounters the human mucosa are of particular interest (4–6). Cells of the innate immunity, including dendritic cells (DCs), express specialized pattern-recognition receptors (PRRs), such as the toll-like receptors (TLRs), that elicit defense mechanisms against infections (7, 8). On DCs, essential antigen-presenting cells, TLR stimulation promotes their maturation, supporting the important role of these receptors in linking innate and adaptive immunity (9–11). Despite their protective function, an inappropriate TLR activation can cause excessive inflammatory responses resulting in immune disorders, such as allergic diseases (12–14). In humans, TLRs are associated with inhalant allergies and airway inflammation. An increased expression of TLRs was observed in the nasal mucosa of patients suffering from allergic rhinitis (15, 16). Moreover, allergenic sources, including pollen-derived compounds and other airborne allergens were described to directly interact with PRRs (4, 17–21).

Although a large body of research investigated the implication of TLRs, particularly TLR4, in allergic airway inflammation (19, 21–24), only few studies focused specifically on pollen sources. The stimulation of epithelial cells by short ragweed pollen (*Ambrosia artemisiifolia*) was shown to result from a TLR4-dependent secretion of TSLP and IL-33, two key alarmins associated with the induction of Th2 responses and allergic sensitization (25). The recruitment of neutrophils investigated in an allergic airway inflammation model induced by short ragweed pollen was also described to depend on TLR4 (26, 27). Kamijo et al. compared DC maturation as well as cytokine secretion in response to stimulation with different pollen species, including Japanese cedar, Japanese cypress, and Kentucky bluegrass and reported that TLR4 was dispensable for most pollen sources except for BP, suggesting a relevant role of the TLR4 pathway in BP-induced DC activation (28). Yet, more concrete studies exploring the importance of TLR4 specifically in BP-induced allergic inflammation as well as the identification of the BP-derived compounds essential for the stimulation of innate immune cells, such as DCs, involved in this process, are still missing.

The existence of pollen-derived compounds, besides allergens, exhibiting an adjuvant activity (29, 30) and the presence of a pollen-inhabiting microbiome (31, 32) suggest that innate immune responses mediated by BP may result from TLR4 agonists derived from microbial contaminations in pollen. The endotoxin lipopolysaccharide (LPS), a cell wall compound of Gram-negative bacteria, represents the most-studied TLR4 ligand due to its strong immunostimulatory effects associated with the onset of inflammatory disorders, including sepsis and airway inflammation (33, 34). In DCs, the signaling cascade induced by the ligation of LPS to TLR4 is well-characterized (35), causing cell activation and secretion of pro-inflammatory cytokines, thus, reflecting its adjuvant property (36, 37). Whether the BP-derived LPS is a determinant for the stimulation of the adaptive immune response is uncertain (38–41). Studies investigating the preferential effector T cell response triggered by LPS are ambiguous, showing that TLR4 signaling promotes the differentiation of different T cell lineages, including Th1, Th2, Th17, and Treg, enhancing or attenuating allergic responses. This discrepancy is likely due to the fact that its immunomodulatory actions depend on several parameters, such as the experimental model, the timing of LPS exposure, and its dose, which varies between allergenic sources and thus influences experimental outcomes (23, 42–46). Apart from LPS, numerous chemically diverse molecules reported to exert immunomodulatory properties via TLR4, such as proteins, glycans, and lipids (47–51), are also commonly found in plants and pollen extracts (52–55). Therefore, it seems plausible that among the myriad of different compounds in the BP matrix, additional adjuvants signaling via TLR4 may exist (56).

Taking into consideration the immunostimulatory activity of BP, its complex composition and the amount of research devoted to TLR4 in allergic inflammation, herein, we examined (i) the role of TLR4 in DC maturation stimulated by birch pollen extracts (BPE), (ii) the extent of the contribution of BPE-derived LPS or other potential TLR4 adjuvant(s) in BPE, and (iii) the relevance of the TLR4-mediated DC activation induced by BPE for the activation of adaptive immune responses. Our findings revealed a major mechanism by which BPE triggers DC activation, and demonstrated the importance of the TLR4 signaling for the subsequent T cell response. As these steps are essential for the initiation of allergic sensitization, the identification of the TLR4 adjuvants in BPE, besides LPS, could provide the basis for the development of specific prophylactic or therapeutic approaches targeting an important pathway by which BP primes allergic inflammation.

## Materials and Methods

### Preparation of Aqueous BPE

Commercial *Betula verrucosa/pendula* pollen (Batch 012517101 Allergon, Angelholm, Sweden) was dissolved in Dulbecco's Phosphate-Buffered Saline (DPBS) (ratio m/v = 0.18 g/ml) and shaken at room temperature (RT) for 1 h. BPE was collected by centrifugation for 15 min at 12,000 g, subsequently filtered through a 0.45 μm syringe filter (Merck Millipore, Merck KGaA, Darmstadt, Germany) and stored at −20°C. A single, carefully characterized BPE batch was used throughout the study. The herein determined levels of TLR4 activity/corresponding endotoxin concentrations were in a similar range to previously published studies using different pollen sources and/or different pollen batches (28, 29, 57, 58).

The total protein concentration was determined using the Bradford assay (Pierce Coomassie Protein assay, Thermo Scientific, Waltham, MA, USA) and a bovine serum albumin (BSA) standard. BPE had a concentration of 2,200 μg/ml. The protein profile was characterized by sodium dodecyl sulfate-polyacrylamide gel electrophoresis (SDS-PAGE) and mass spectrometry, as described (59).

### Bet v 1 Quantification

An in-house sandwich ELISA was performed to quantify the Bet v 1 content in BPE. The monoclonal anti-Bet v 1.0101 antibody was used for coating and detection was done with an affinity-purified polyclonal rabbit anti-Bet v 1.0101 antibody in combination with a horseradish peroxidase (HRP)-conjugated anti-IgG/IgM antibody. Based on a standard curve with an in-house produced recombinant Bet v 1 (isoform 1.0101), termed Bet v 1 in the following, and using linear regression, 0.125 mg/ml Bet v 1 was calculated per mg/ml of BPE (12.5% of total protein content) (29, 60).

### Lipopolysaccharide (LPS)

Pure LPS derived from *E.coli* O111:B4 (L3024-10MG, Sigma-Aldrich, Darmstadt, Germany) or ultrapure LPS from *Salmonella minnesota* (tlrl-smlps, Invivogen, San Diego, CA, USA) were used in the different screening systems.

### TLR4 Human Embryonic Kidney (HEK) Reporter Cell Assay

The capacity of BPE and LPS to activate the TLR4 signaling pathway was assessed *in vitro* using HEK-Blue^TM^ cell lines stably transfected with murine or human TLR4 (hkb-mtlr4, SEAP Reporter 293 cells; hkb-htlr4, SEAP Reporter 293 cells, Invivogen, San Diego, CA, USA). Cells were treated with serial dilutions of LPS and BPE for 24 h, at 37°C and 5% CO_2_. The activity of the alkaline phosphatase was determined in the cell culture supernatants via a colorimetric enzyme assay using QUANTI-Blue^TM^ (Invivogen, San Diego, CA, USA). Optical density (OD) was measured at 650 nm. To evaluate potential treatment-associated cytotoxic effects, cell viability was assessed using the colorimetric MTT (3-(4,5-dimethylthiazol-2-yl)-2,5-diphenyltetrazolium bromide) assay (Sigma, Saint Louis, USA). Because the signal induced by BPE in the murine (m)TLR4 HEK assay reflected the total activation of the TLR4-NFkB pathway, the TLR4 activity mediated by LPS was indirectly determined via interpolation. Linear regression based on the LPS standard curve and a titration of BPE was used to estimate the amount (μg) of LPS per mg of total protein in BPE.

### Recombinant Factor C (rFC) Assay

As second method for LPS quantification, the PyroGene^TM^ Recombinant factor C Endpoint Fluorescent Assay (50-658U, Lonza, Basel, Switzerland) was used following the manufacturer's instructions. Briefly, serial dilutions of the samples (200–0.1 μg/ml) and LPS (1,000–0.4 ng/ml) were prepared in duplicates in endotoxin free ultra-pure water in a 96-well microplate and pre-incubated for 10 min at 37°C in a plate reader (Tecan i-control 1.7.1.12, Tecan Trading AG, Switzerland). The working solution comprising the fluorogenic substrate, the assay buffer and the enzymes were mixed in a ratio 5:4:1 (v/v) and distributed into the pre-warmed microplate. Fluorescence intensity was measured at 380/440 nm after 60 min at the optimal gain. For quantification, blank values (buffer only) were removed, and a linear regression was calculated based on the LPS standard curve to obtain the amount (μg) of LPS per mg of total protein in the sample.

The average of the results obtained in both quantification methods was taken to determine the value of “nLPS,” defined here as the relative amount of LPS contained in BPE, and used in the study to compare the effects of BPE vs. BPE-derived LPS.

### Protein Removal

Beads coated with proteinase K from *Tritirachium album* (P9290-10UN, Sigma-Aldrich Darmstadt, Germany) were used to digest proteins in BPE. A volume of 500 μl of BPE (2,200 μg/ml) was mixed with one unit of proteinase K beads and incubated for 24 h at RT on a shaker (Kenshi Co, LTD, Japan). To stop the digestion, the beads were removed by centrifugation (5 min, 3,250 g) to collect the supernatant, which was termed “protein-free BPE” (P3BPE). As control, the same procedure was repeated with 1x PBS and uncoupled sepharose control beads recommended by the company (Pharmacia Biotech, Sepharose CL-4B, Uppsala, Sweden).

### Lipid Removal

To remove the lipids in BPE the Cleanascite^TM^ solution (X2555-10, Biotech Support, Monmouth Junction, USA) was used and the respective protocol was adapted according to manufacturer's recommendations. A volume of 250 μl of BPE was incubated for 10 min with the Cleanascite^TM^ solution in a ratio 1:1 (v/v). After centrifugation (5 min, 16,000 g), the supernatants of BPE fractions were collected. The same procedure was repeated using nLPS and PBS as controls.

The fractions derived from both the protein and the lipid removal experiments were analyzed via SDS-PAGE and examined regarding their TLR4-activating capacity compared to the untreated BPE and nLPS using the mTLR4 HEK assay.

### LPS Removal *via* Polymyxin B- (PMB-) Pull-Down

A pull-down assay was established with PMB-coated agarose beads (P1411, Sigma, Saint Louis, USA) in order to remove PMB-binding components, especially LPS, from BPE. The beads were washed with 1x PBS, then blocked with 1x PBS containing 3% BSA overnight at RT in order to avoid non-specific binding to the bead matrix. After five washing steps, 800 μl of BPE (80 μg/ml) or LPS (100 ng/ml) were incubated with the beads for 2 h at RT. The supernatant was collected after centrifugation (5 min, 16,000 g). As negative control, BPE or LPS were incubated with uncoupled sepharose beads (Pharmacia Biotech, Sepharose CL-4B, Uppsala, Sweden). The supernatants (unbound fractions) were compared to untreated BPE or LPS, respectively, in the TLR4 HEK assay.

### Culturing of Murine Bone Marrow-Derived Dendritic Cells (BMDCs)

Experiments were performed using BM cells from both male and female wild-type (WT) or TLR4-knock-out (TLR4-KO) C57/BL6 mice (Jackson Laboratory, Maine, USA) or WT Balb/c mice maintained under specific pathogen-free conditions at the Animal Facility of the University of Salzburg and used above 8-weeks of age. All experiments were approved under a Project License granted by the Austrian Federal Ministry (BMWF-66-012/0015-WF/V/3b/2015) and conducted in accordance with the local guidelines.

The isolation was conducted as described previously (61). Briefly, the bone marrow was isolated from the femora and tibia under sterile conditions. Cells were cultured in Petri dishes at a density of 4 × 10^6^ cells/dish for 8 days in 20 ml RPMI medium (Sigma) supplemented with 5% (v/v) heat-inactivated fetal calf serum (iFCS) (Sigma), 2 mM L-glutamine (Sigma), 1% penicillin-streptomycin, 200 μM β-mercaptoethanol, and 10 ng/ml recombinant mouse granulocyte-macrophage colony-stimulating factor (rmGM-CSF, Immunotools, Germany). A concentration of 10 ng/ml was chosen to minimize the alongside generation of macrophages (62). At day 3, additional 10 ml of medium containing rmGM-CSF was added, and at day 6, half of the medium was exchanged for fresh medium containing rmGM-CSF. At day 8, only the non-adherent cells, enriching the DC population rather than the adherent macrophages, were harvested and either used fresh or kept frozen in Cryostor (Stemcell Technologies, Bothell, WA, USA) until use. The herein termed BMDCs consisted of a mixed population of DCs and macrophages, both relevant antigen-presenting cells (63).

### *In vitro* BMDC Activation Assay

Cells were seeded in 96-well U-bottom microplates (Greiner Bio-One, Frickenhausen, Germany) at a density of 2 × 10^5^ cells per well and stimulated with BPE or the corresponding nLPS concentration for 24 h at 37°C, 5% CO_2_. BMDCs derived from mice with a C57/BL6 genetic background were stimulated with 20 μg/ml BPE and 2.6 ng/ml nLPS, whereas for Balb/c BMDCs, 60 μg/ml BPE and 7.8 ng/ml nLPS were used. The TLR2 agonist, FSL-1 (Invivogen, San Diego, USA), was used as positive control (100 ng/ml) for the responsiveness of TLR4-KO cells, and medium was used as negative control. The surface expression of the co-stimulatory molecules, CD40, CD80, CD86, and markers favoring type 2 immune responses, OX40L and Jagged-1, were analyzed. Culture supernatants were also collected in order to analyze the secreted cytokine profile via flow cytometry (Cytoflex S, Beckman Coulter, CA, USA) using a customized LEGENDplex™ kit including interleukin (IL)-23, IL-33, MCP-1, IL-12p70, IL-6, IL-1β, TNF-α, IL-1α, TGF-β, IFN-γ, IL-2, IL-10, and IL-17A (BioLegend, CA, USA). To investigate the regulation of TLR4 surface expression, C57/BL6 BMDCs were stimulated with 20 μg/ml BPE and 2.6 ng/ml nLPS for 24, 18, 12, 8, 4, 2, and 1 h at 37°C, 5% CO_2_. After stimulation, the cells were washed with DPBS, then stained with the eBioscience™ FixableViability Dye eFlour™506 (Invitrogen, San Diego, CA, USA) (diluted 1:1,000 in 100 μl). Unspecific binding of dyes was blocked with the supernatant from the hybridoma cell line 2.442 (ATCC HB-197) producing anti-mouse CD16/CD32 IgG antibodies for 5 min at 4°C. Cells were stained for 30 min at 4°C with 100 μl per well of antibody mixtures containing PE anti-mouse CD11c (clone N418; eBioscience™, San Diego, CA, USA), APC/Cy7 anti-mouse CD86 (clone GL-1; BioLegend), PerCP/Cy5 anti-mouse CD40 (clone 3/23; BioLegend) and BV421 anti-mouse CD80 (clone 16-10A1; BioLegend) and APC anti-mouse CD339 (Jagged-1) (clone HMJ1-29, BioLegend) and PE/Dazzle™ 594 anti-mouse CD252 [OX40 ligand (OX40L)] antibodies (clone RM134L, BioLegend). All antibodies were diluted 1:1,000, except for CD40, which was diluted 1:250. For the analysis of TLR4 surface expression, APC anti-mouse CD11c (N418, eBioscience™) and PE/Cy7 anti-mouse TLR4 (clone SA15-21, BioLegend) antibodies were used, both diluted 1:100. Stained cells were washed twice with FACS buffer (1x PBS, 0.5% BSA, 2 mM EDTA) and measured via flow cytometry (Cytoflex S, Beckman Coulter). For data analysis, the FlowJo software (FlowJo, LLC, Ashland, Oregon, USA) was used. Gating strategies are represented in Supplementary Figure 7.

### *In vitro* Inhibition of BMDC Activation Using PMB or a TLR4 Antagonist

Cells were pretreated with 0.01, 0.1, and 1 μg/ml soluble PMB (tlrl-PmB, Invivogen, San Diego, USA) for 1 h at 37°C and 5% CO_2_ prior to the stimulation with 20 μg/ml BPE or 2.6 ng/ml nLPS for 24 h at 37°C, 5% CO_2_. In a similar approach, ultrapure LPS-RS (tlrl-prslps, Invivogen, San Diego, USA) a penta-acetylated lipid A structure derived from the photosynthetic bacterium *Rhodobacter sphaeroides* was used to competitively antagonize the TLR4 receptor activation without interacting with TLR2. Cells were incubated with a ratio nLPS:TLR4 antagonist 1:1, 1:10, 1:100, 1:1,000 in Balb/c BMDCs (equivalent to 0.0078, 0.078, 0.78, and 7.8 μg/ml of TLR4 antagonist) for 1 h at 37°C, 5% CO_2_ before stimulation with 60 μg/ml BPE or 7.8 ng/ml nLPS for 24 h at 37°C, 5% CO_2_.

### *In vitro* Co-culture of Pulsed-BMDCs and Bet v 1-Specific Hybridoma T Cells

WT Balb/c BMDCs were seeded at a density of 40,000 cells per well in a U-bottom 96 well microplate and stimulated for 24 h at 37°C, 5% CO_2_ with either 100, 80, 60, or 40 μg/ml of BPE or with the corresponding concentration of Bet v 1 (12.5, 10, 7.5, or 5 μg/ml) as determined by sandwich ELISA. BPE-stimulated BMDCs (80 μg/ml) were also compared to BMDCs treated with Bet v 1 and nLPS in the relative concentrations measured in BPE (10 μg/ml and 10.4 ng/ml, respectively). In addition, BPE pre-incubated with PMB, and BMDCs pre-treated with TLR4 antagonist, were used to investigate the TLR4-dependent DC activation. Pulsed BMDCs were washed twice with DPBS before hybridoma CD4-positive (+) T cells specific for the immunodominant T cell epitope of Bet v 1 (epitope 142–153) were added at a density of 400,000 cells per well (ratio 1:10 BMDC/T cells) (60). The Bet v 1-specific hybridoma cells were kindly provided by Prof. Richard Weiss from the University of Salzburg. The medium for the hybridoma cells was composed of DMEM high glucose medium (Sigma-Aldrich, Darmstadt, Germany) supplemented with 4 mM L-glutamine, 10% iFCS and antibiotics penicillin and streptavidin (1 mg/ml and 1X, respectively). After 24 or 48 h of incubation at 37°C, 5% CO_2_, the secretion of IL-2 as an indicator for T cell proliferation was analyzed in the cell culture supernatants via ELISA (Invitrogen, San Diego, USA) as described previously (60).

### Human Monocyte-Derived Dendritic Cells (moDCs)

Peripheral blood mononuclear cells (PBMCs) were isolated by density gradient centrifugation (Lymphoprep, Axis-Shield) from fresh blood of allergic and non-allergic volunteers selected for eligibility at the Chair and Institute for health sciences at Klinikum Augsburg, UNIKA-T, in Germany. Supplementary Table 1 summarizes the demographic information and the sensitization profile of the donors. “Non-allergic” blood donors had total serum IgE ≤ 100 kU/L and no clinical history of respiratory allergies. “Allergic” blood donors had doctor-diagnosed or self-reported seasonal allergic rhinitis, with symptoms in springtime, and elevated levels of specific IgE (ImmunoCAP class ≥2) toward BP (donor 5 and 7), grass pollen (donor 5, 6, and 7) or house dust mite (donor 3, 4, and 5). All samplings were done outside of the birch pollen season, and none of the donors was undergoing allergen immunotherapy treatment. The study was approved by the technical university of Munich (ethics code: 54/17 S).

CD14+ monocytes were isolated from PBMCs by MACS separation on an AutoMACS^TM^ device (Miltenyi Biotec, Bergisch Gladbach, Germany) and cultured in RPMI medium completed with 5% iFCS, 2 mM L-Glutamine, 1% gentamycin and 50 μM β-mercaptoethanol at 37°C, 5% CO_2_ in the presence of 500 U/ml rhGM-CSF and 50 U/ml rhIL-4 (Promocell, Heidelberg, Germany). On day 5, immature DCs were harvested, seeded at a density of 10^5^ cells per well in a 96-well flat-bottom plate and stimulated with serial dilutions of BPE or nLPS for 24 h at 37°C, 5% CO_2_. For the TLR4 inhibition approach, the moDCs were pretreated with 260 ng/ml TLR4 antagonist (tlrl-rslps; Invivogen, Toulouse, France) for 1 h and then stimulated with 2 μg/ml BPE or 0.26 ng/ml nLPS for 24 h at 37°C, 5% CO_2_. For analysis of the maturation markers and the TLR4 receptor surface expression, the cells were washed with DPBS and stained with the viability dye PF840 (Promocell) for 15 min at 4°C. Unspecific antibody binding was blocked by incubating the cells with rFc blocking reagent (Miltenyi Biotech) for 5 min at RT. Cells were stained for 30 min at 4°C in the dark with an antibody cocktail consisting of PE/Cy anti-human CD1a (clone HI149, BioLegend), PE anti-human CD40 (clone 5C3, eBioscience™), PE/CF594 anti-human HLA-DR (clone G46.6, BD Biosciences), APC anti-human CD83 (clone HB15e, BD Pharmingen), APC/H7 anti-human CD80 (clone CD28.2, BD Biosciences), Pacific Orange anti-human CD86 (clone BU63, ExBio) and FITC anti-human CD197 (CCR7) (clone 3D12, BD Biosciences) or with PE/Cy anti-human CD1a and AF700 anti-human CD284 (TLR4) (cloe HTA-125, eBioscience™). After washing the cells with FACS buffer (DPBS, 2 mM EDTA, 5% iFCS), measurements were performed on a Cytoflex LX device (Beckman Coulter) and data were analyzed using the Kaluza software (Beckman Coulter).

### *In vivo* Immunization Model

Female or male IL-4/eGFP-enhanced transcript (4get) mice (Jackson laboratory, Bar Harbor, Me, USA), at 20–30-weeks of age, were immunized without additional adjuvants, as described previously (29). Intradermal injection was performed with either 65 μg BPE (*n* = 8) or with BPE pre-incubated for 1 h with 3.5 μg PMB (ratio 20:1 BPE) (*n* = 8) or with PMB alone as control (*n* = 6). The amount of PMB was below the published toxic level (64). Samples were prepared freshly and sterile filtered (0.22 μm) before use. After 5 days, mice were sacrificed, and the lymph nodes were harvested for flow cytometry analysis. Skin-draining inguinal lymph nodes of the mice were collected in PBS, and lymphocytes were stained with an APC anti-mouse CD4 antibody (clone RM4-5, BioLegend) at a dilution of 1:200 in FACS buffer and analyzed for IL-4/eGFP expression using flow cytometry. Cell viability was also verified using the SYTOX™ Red Dead Cell Stain (ThermoFisher™, Oregon, USA). The same number of cells (50,000 events, excluding debris) was recorded per mouse and the frequency of GFP/IL-4+ among CD4+ cells was analyzed. *In vivo* experiments were performed according to national guidelines approved by the Austrian Federal Ministry (BMBWF - V/3b; approval number 2021-0.118.574).

### Statistics

Statistical analysis was performed using Student's *t*-tests, One-Way or Two-Way ANOVA with *post-hoc* Dunnett's, Sidak's, or Tukey's multiple comparison tests. For the time curve, a smoothening of second order (four neighbors) was defined. The data represent the mean and the standard deviation (SD) of duplicate or triplicate measurements. The confidence interval was at 95%. Statistical significance is indicated for *p* < 0.05 (^*^), *p* < 0.01(^**^), *p* < 0.001 (^***^) and *p* < 0.0001 (^****^). All *in vitro* experiments were performed at least three times. GraphPad Prism 9 was used for data analysis.

## Results

### Evaluation of LPS Content in BPE

**Figure 1:**
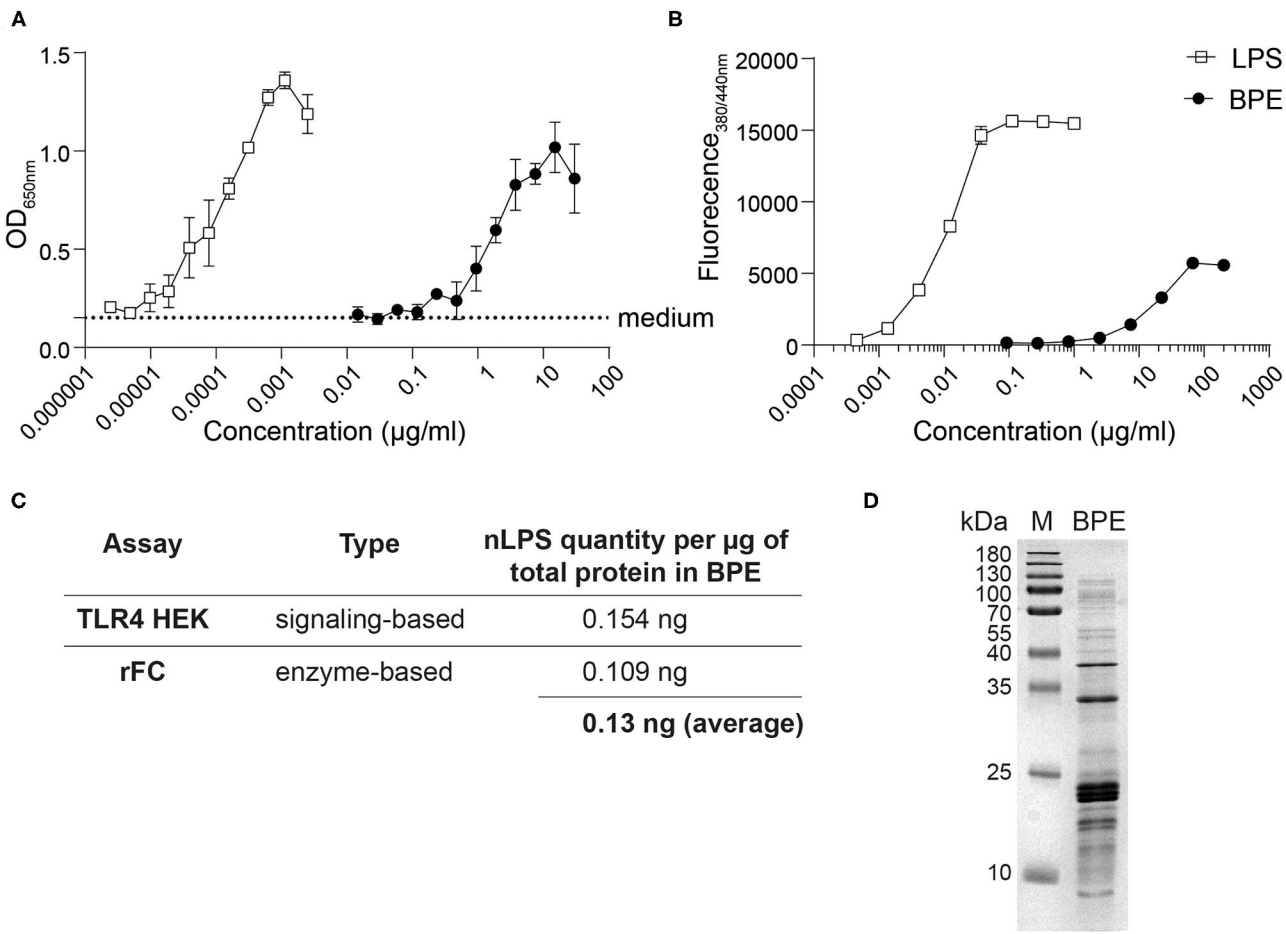
Relative quantification of the LPS content in BPE. Titration curves of BPE (based on total protein concentration) and an LPS standard were performed using the mTLR4 HEK reporter assay **(A)** and the rFC endotoxin detection assay **(B)** in order to quantify the relative endotoxin level in BPE, using linear regression (Supplementary Figure 1). Dotted line represents the average of medium-treated cells in **(A)**. Assays were performed in duplicates and repeated at least twice. Summary table showing the average amounts of nLPS determined in BPE for each method and the average of both approaches combined **(C)**. Coomassie Brilliant Blue-stained SDS-PAGE gel of BPE **(D)**.

For the relative quantification of LPS in BPE, two complementary methods were used: (i) HEK reporter cells transfected with either the murine (Figure 1A) or human TLR4 (data not shown), and (ii) a rFC endotoxin detection assay based on the endotoxin's capacity to activate the recombinantly produced factor C enzyme (Figure 1B). Stimulation with BPE resulted in a dose-dependent activation of the TLR4-NFκB signaling pathway and the rFC activity, respectively. To determine the relative amount of LPS per total protein content in BPE, a standard curve of pure LPS was used in both methods for interpolation by linear regression (Supplementary Figure 1). This calculated amount of LPS “naturally” present in BPE was termed “nLPS.” An average of 0.13 ng nLPS per μg of total protein in BPE (Figure 1C), corresponding to a ratio of ~1:10,000, was determined and used for the following stimulations. The integrity of our BPE was verified by SDS-PAGE, showing the complete protein profile within the extract (Figure 1D).

### BPE-Induced TLR4 Signaling and Dendritic Cell Activation Are Blocked by Polymyxin B

**Figure 2:**
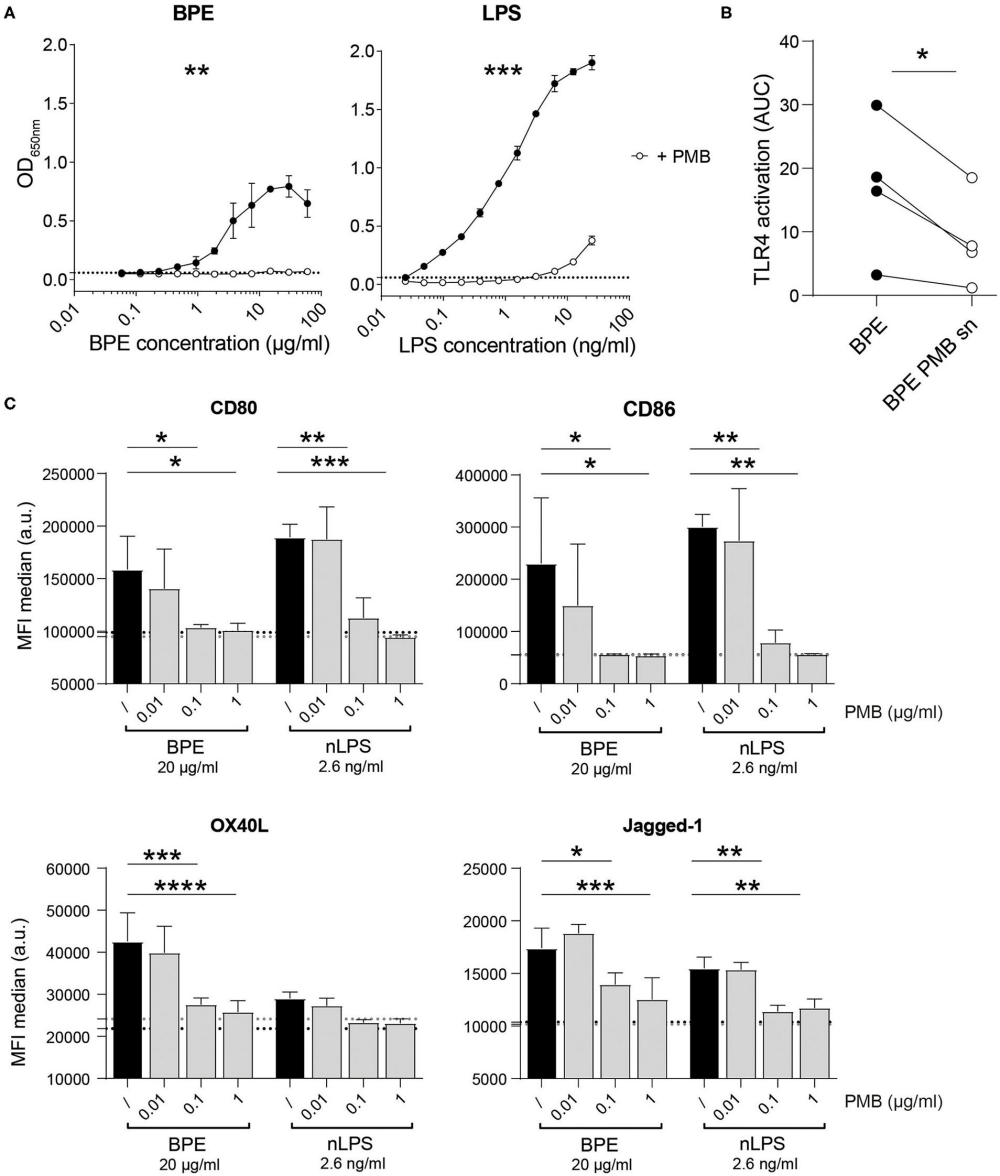
BPE- and nLPS-induced TLR4-NFκB signaling and murine BMDC activation are inhibited by PMB. Murine TLR4 HEK-blue reporter cells were treated with 5 μg/ml PMB 30 min prior to stimulation with serial dilutions of LPS and BPE **(A)**. Comparison of the TLR4 signaling activity induced by BPE before and after (BPE PMB sn) the PMB-pull down assay **(B)**. Data represent the area under the curve (AUC) of the results of four individual mTLR4 HEK assays. The expression of the surface markers CD80, CD40, OX40L, and Jagged-1 was analyzed on C57/BL6 BMDCs pretreated for 1 h with 0.01, 0.1, or 1 μg/ml of soluble PMB (gray bars) or untreated (/, black bars) before stimulation with 20 μg/ml BPE or 2.6 ng/ml nLPS. Black and gray dotted lines represent the average of medium- or PMB-treated cells (highest PMB concentration, 1 μg/ml), respectively. The BMDC activation assays were performed in triplicates. Results are representative of three individually performed experiments **(C)**. Statistics were calculated using either an un-paired Student's *t*-test on the area under the curve (AUC) obtained from duplicate measurements **(A)**, a paired Student's *t*-test **(B)**, or a Two-Way ANOVA with a Dunnett's multiple comparisons test **(C)**.

To address the contribution of LPS to the immune stimulation mediated by BPE, the antibiotic PMB, which neutralizes the biological activity of LPS by binding to the lipid A part of the endotoxin (65), was used as inhibitor in the mTLR4 HEK reporter system as well as in the BMDC activation assay. The dose-dependent TLR4-NFkB signaling induced by BPE and pure LPS was significantly abrogated by the addition of soluble PMB prior to stimulation (Figure 2A). The PMB concentration was established by titrating the antibiotic in BPE- or LPS-stimulated mTLR4 HEK reporter cells; for 5 μg/ml PMB a complete inhibition was observed (Supplementary Figure 2A). In addition, agarose beads coated with PMB were used in an attempt to remove LPS from BPE via a pull-down strategy (Supplementary Figure 2B). The supernatant containing the unbound fraction of BPE (BPE PMB sn) was analyzed regarding its capacity to activate TLR4 in the HEK reporter assay (Figure 2B). Compared to untreated BPE, the TLR4 signal induced by the supernatant of BPE from the PMB-pull-down (BPE PMB sn) was significantly lower. For proof of concept, the PMB-pull-down was also performed with pure LPS and resulted in a similar reduction (Supplementary Figure 2D). The PMB-pull-down assays completed with the uncoupled control beads did not modify the TLR4 signaling induced by BPE or LPS, excluding the non-specific binding of BPE- or LPS-compounds to the bead matrix (Supplementary Figures 2C,D).

The capacity of either BPE or the related amount of nLPS to activate DCs *in vitro* was analyzed by stimulating BMDCs in the presence of different PMB concentrations (Figure 2C). Both BPE and nLPS enhanced efficiently the surface expression of the co-stimulatory molecules, CD80 and CD86, as well as the Th2-associated markers, OX40L and Jagged-1, which was completely abolished by PMB at 0.1 μg/ml. In addition, several inflammatory and Th-associated cytokines secreted into the cell culture supernatant were monitored (Supplementary Figure 3). Most cytokines analyzed, including the pro-inflammatory IL-6, IL-1α, IL-1β, TNFα, MCP-1, IL-33, and IL-17A, the Th1-associated cytokines IFN-γ and IL-12p70, the regulatory cytokine IL-10, and the Th2/Th17-associated IL-23 were significantly reduced by PMB in a dose-dependent manner. IL-2 and TGF-β were not significantly induced by BPE stimulation.

### BPE-Derived Lipids, but Not Proteins, Contribute to the BPE-Induced TLR4 and BMDC Activation

**Figure 3:**
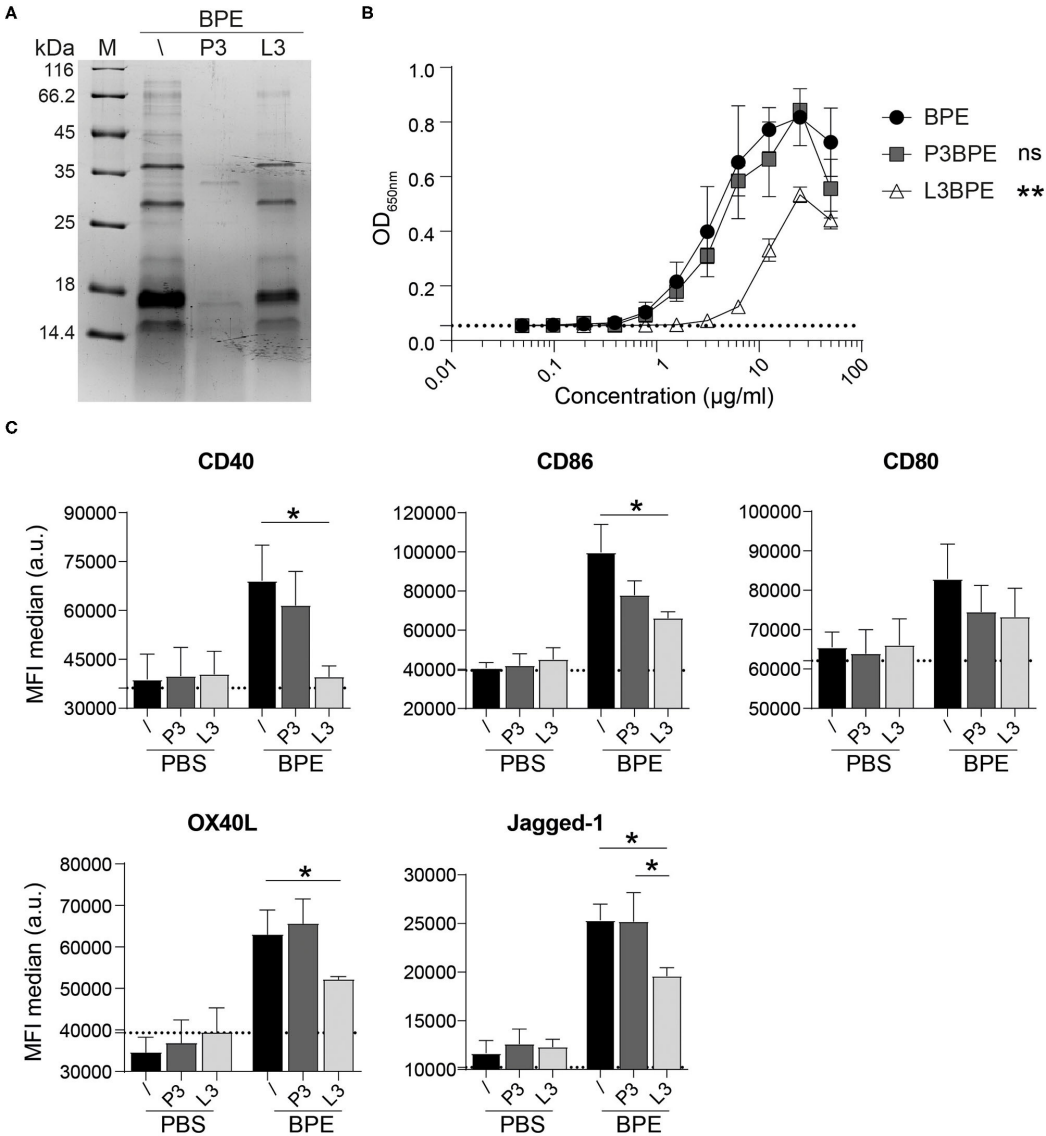
BPE-derived lipids rather than proteins contribute to the BPE-induced TLR4 signaling and BMDC activation. Coomassie Brilliant Blue-stained SDS-PAGE visualization of untreated (/), protein-free (P3), and lipid-free (L3) fractions of BPE **(A)**. The TLR4 signaling capacity of P3BPE and L3BPE compared to untreated BPE was analyzed in the mTLR4 HEK assay **(B)**. For statistical analysis, a One-Way ANOVA of the AUC with a Dunnett's multiple comparisons test was used comparing the fractions with untreated BPE. Surface expression of the costimulatory molecules CD40, CD86, CD80, and the Th2-associated markers OX40L and Jagged-1 was analyzed in Balb/c BMDCs upon stimulation with BPE (60 μg/ml), P3BPE, L3BPE, or the corresponding PBS controls **(C)**. Dotted lines represent the average of medium-treated cells. Statistics were computed by a One-Way ANOVA with a Tukey's multiple comparisons test.

Assuming that the TLR4-dependent immunostimulatory activity of BPE might not be solely restricted to the effects of LPS, the BPE composition was further investigated by generating either protein- or lipid-depleted fractions thereof, termed “protein-free” (P3) and “lipid-free” (L3) fraction, respectively (Supplementary Figure 4A). The protein profile of the BPE fractions was analyzed via SDS-PAGE to verify that proteins were efficiently degraded in P3BPE or remained intact in the L3BPE fraction (Figure 3A). A 24 h incubation with proteinase K resulted in a complete digestion of proteins in BPE (Supplementary Figure 4B). Compared to untreated BPE, P3BPE was still able to trigger TLR4 signaling in the HEK reporter assay, whereas L3BPE significantly lost its TLR4 activation capacity (Figure 3B). The PBS buffer, used for the aqueous extraction, treated with the same fractionation protocols, did not induce any signal in the TLR4 HEK assay (Supplementary Figure 4C) as well as in the BMDC activation assay (Figure 3C), excluding, one the one hand, a possible external LPS contamination from the extraction procedure and, on the other hand, a non-specific signal induced by the fractionation strategies themselves. The bead matrix also had no influence on the readout, as confirmed by the assay performed with the uncoupled control beads (Supplementary Figure 4D). In contrast, nLPS treated with the lipid removal reagent still fully activated TLR4; no difference was observed compared to the untreated nLPS (Supplementary Figure 4E).

Likewise, the degradation of proteins in BPE (P3BPE) did not alter its capacity to activate BMDCs compared to untreated BPE, as measured by the enhanced expression of CD40, CD86, and CD80 as well as of OX40L and Jagged-1. In contrast, upon lipid-removal, L3BPE was significantly less potent in inducing the surface expression of CD40, CD86, OX40L, and Jagged-1, whereas the regulation of CD80 was not significantly changed (Figure 3C). Similar results were obtained with C57/BL6-derived BMDCs (data not shown). A similar pattern was observed for the secretion of IL-6, IL-1α, IL-1β, TNFα, IL-12, IL-23, and IL-33, analyzed in the cell culture supernatants (Supplementary Figure 4F). Non-specific treatment-associated and cytotoxic effects were ruled out by including PBS as control (Figure 3C, Supplementary Figures 4C,F) and performing cell viability staining (data not shown), respectively. Neither the protein digestion nor the lipid extraction procedure affected cell activation. Together, these data indicate that BPE-derived lipids rather than proteins account for the BPE-induced TLR4 signaling and BMDC activation.

### BPE-Induced Activation of Murine Dendritic Cells Is TLR4-Dependent

**Figure 4:**
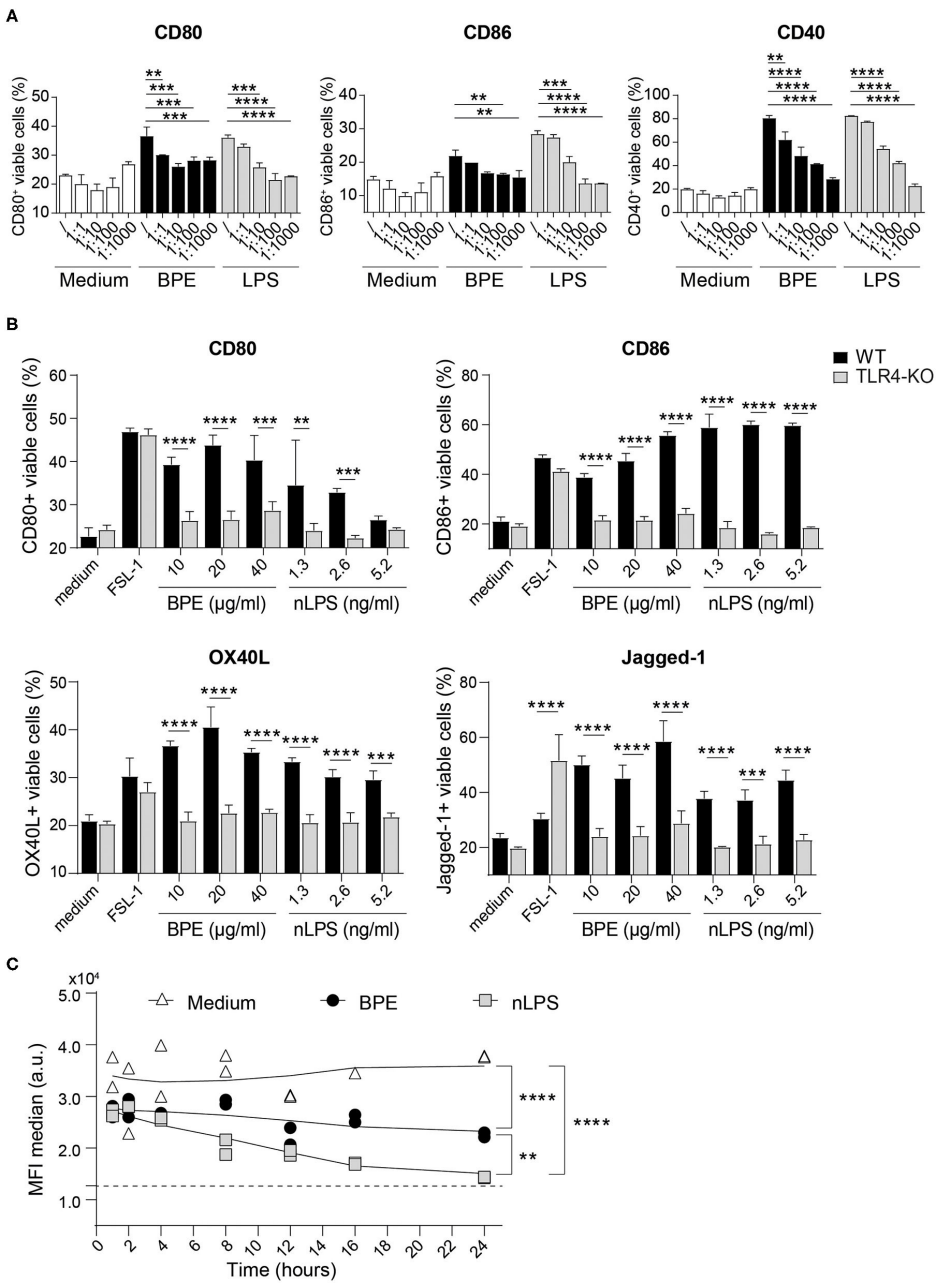
BPE-induced BMDC activation is TLR4-dependent. Surface expression of the co-stimulatory molecules CD80, CD86, and CD40 was analyzed in Balb/c BMDCs pretreated with different concentrations of the TLR4 antagonist, ultra-pure LPS-RS, before stimulation with 60 μg/ml BPE, the corresponding nLPS concentration of 7.8 ng/ml or untreated (medium) **(A)**. The ratios nLPS to TLR4 antagonist correspond to increasing doses of the antagonist in relation to constant nLPS (/ = no antagonist; 1:1 = 7.8 ng/ml, 1:10 = 78 ng/ml, 1:100 = 0.78 μg/ml and 1:1,000 = 7.8 μg/ml). A Two-Way ANOVA with a Dunnett's multiple comparisons test was used for statistical comparison of the reference (without TLR4 antagonist) with all other samples among treatment groups (medium, BPE or LPS). Surface expression of activation and Th2-associated markers was examined on WT vs. TLR4-KO C57/BL6 BMDCs treated with either 10, 20, and 40 μg/well BPE, 1.3, 2.6, and 5.2 ng/ml nLPS, 100 ng/ml FSL-1 or with medium (untreated condition) **(B)**. A Two-Way ANOVA with a Sidak's multiple comparisons test comparing WT and TLR4-KO cells. Analysis of TLR4 surface expression in C57/BL6 BMDCs stimulated with either 20 μg/ml BPE or the corresponding nLPS concentration of 2.6 ng/ml over a period of 24 h **(C)**. The assay was repeated three times in total. Dotted line represents the MFI baseline determined with the TLR4-KO BMDCs. Data are shown as smoothed trend line for each treatment condition (smoothening function of 2nd order, 4 neighbors). Statistics were calculated using a Two-Way ANOVA with a Tukey's multiple comparisons test.

In the second major part of this study, the function of TLR4 regarding the activation of BMDCs mediated by BPE was addressed in more depth. To do so, the TLR4 signaling was blocked using a TLR4 antagonist, the ultrapure LPS-RS, which resulted in a dose-dependent suppression of BPE- and nLPS-induced up-regulation of CD80, CD86, and CD40 molecules (Figure 4A). A significant and complete inhibition was achieved when the antagonist was added at a 100- and 1,000-fold excess in relation to nLPS (7.8 ng/ml). In contrast to WT cells, TLR4-deficient cells failed to up-regulate the surface markers CD80, CD86, OX40L, and Jagged-1 in response to BPE or nLPS. The overall responsiveness of TLR4-KO BMDCs despite their KO phenotype was confirmed using the bacterial TLR2 agonist, FSL-1, for cell stimulation (Figure 4B, Supplementary Figure 5A). Additionally, the TLR4 surface expression in BMDCs was monitored over a period of 24 h after stimulating the cells with either 20 μg/ml BPE or the equivalent nLPS concentration of 2.6 ng/ml. Both BPE and nLPS elicited a progressive and significant down-regulation of the TLR4 surface expression in BMDCs compared to medium-treated cells; the effect observed for nLPS was more pronounced than for BPE (Figure 4C, Supplementary Figure 5B).

### BPE-Mediated Activation of Human moDCs Depends on TLR4 Signaling

**Figure 5:**
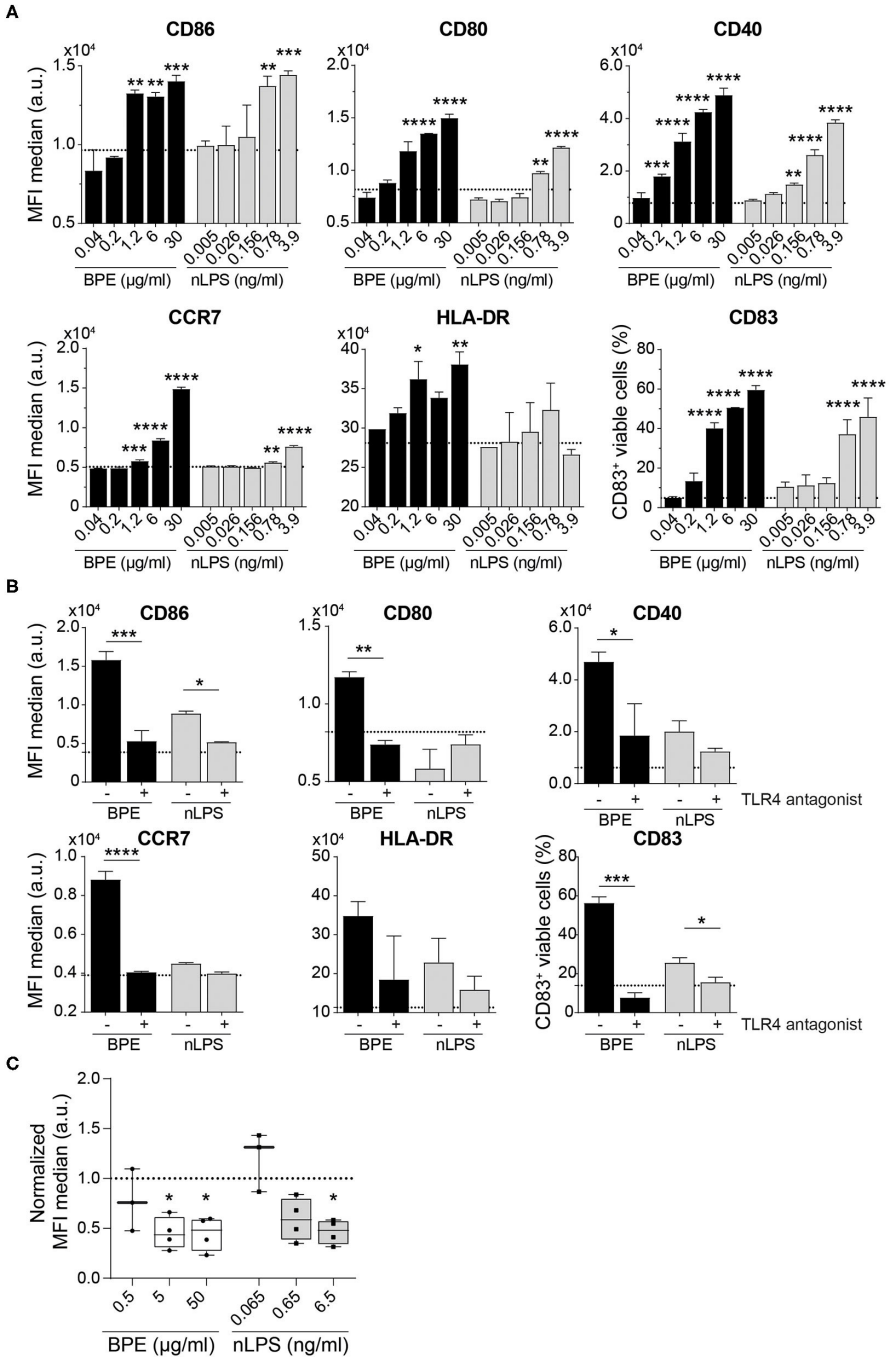
BPE-induced activation of human moDCs is abolished using a TLR4 antagonist. Surface expression of six distinct maturation markers on human moDCs was analyzed upon treatment with serial dilutions of BPE or the related nLPS concentration **(A)**. Regulation of the maturation markers in human moDCs stimulated with 2 μg/ml BPE or 0.26 ng/ml nLPS after administration of 260 ng/ml TLR4 antagonist (ratio 1:1,000 of nLPS to TLR4 antagonist) was analyzed **(B)**. Dotted lines represent the average of medium-treated cells. Representative data of two single non-allergic donors are shown in **(A,B)**. Each assay was repeated with three donors (allergics and non-allergics). Statistics were calculated using a Two-Way ANOVA with a Dunnett's and Sidak's multiple comparisons test, respectively. Surface expression of TLR4 after 24 h stimulation with different BPE or corresponding nLPS concentrations **(C)**. Normalized data derived from four independently performed experiments (*n* = 4 allergic donors) are shown. The MFI of the data was normalized toward the untreated/medium-treated control (dotted line). For statistics, the untreated reference was compared to treated samples with a mixed-effect analysis with a Dunnett's multiple comparison test.

The human relevance of the findings obtained from our murine BMDC model was further examined *in vitro* using human moDCs. The immunostimulatory activity of BPE was demonstrated by the up-regulation of the maturation markers CD86, CD80, CD40, HLA-DR, and CD83 on moDCs as well as of the chemokine receptor CCR7, required for migration of the DCs toward the draining lymph nodes as prerequisite for T cell activation (Figure 5A); however, in this human model, the DC activation induced by nLPS was weaker compared to BPE. Pretreatment of the cells with the TLR4 antagonist fully inhibited the activation of moDCs induced by both BPE and the corresponding nLPS concentration (Figure 5B). The concentration of the TLR4 antagonist needed to achieve a complete inhibition was established in a titration experiment using moDCs stimulated with 100 ng/ml LPS, which was reached at a 1,000-fold excess of the antagonist (Supplementary Figure 6A). Non-specific modulatory effects of the TLR4 antagonist were ruled out by treating the cells only with the TLR4 antagonist (Supplementary Figure 6B). Similar to the murine DC experiments, the TLR4 surface expression decreased in a dose-dependent manner after 24 h stimulation with BPE compared to untreated cells from allergic donors. Here, stimulation with nLPS resulted in a similar pattern as BPE (Figure 5C, Supplementary Figure 6C). The regulation of the TLR4 expression was not different between the moDCs from the BP allergic donor compared to the moDCs from donors allergic to other sources, including house dust mite or grass pollen, suggesting a general internalization mechanism of the receptor in response to pro-inflammatory stimuli.

### BPE-Induced TLR4-Mediated DC Activation Influences T Cell Responses *in vitro* and *in vivo*

The final part of the study aimed to assess the relevance of the BPE-induced and TLR4-mediated DC activation for the adaptive T cell response, an essential step succeeding the innate immune activation in the process of allergic sensitization.

**Figure 6:**
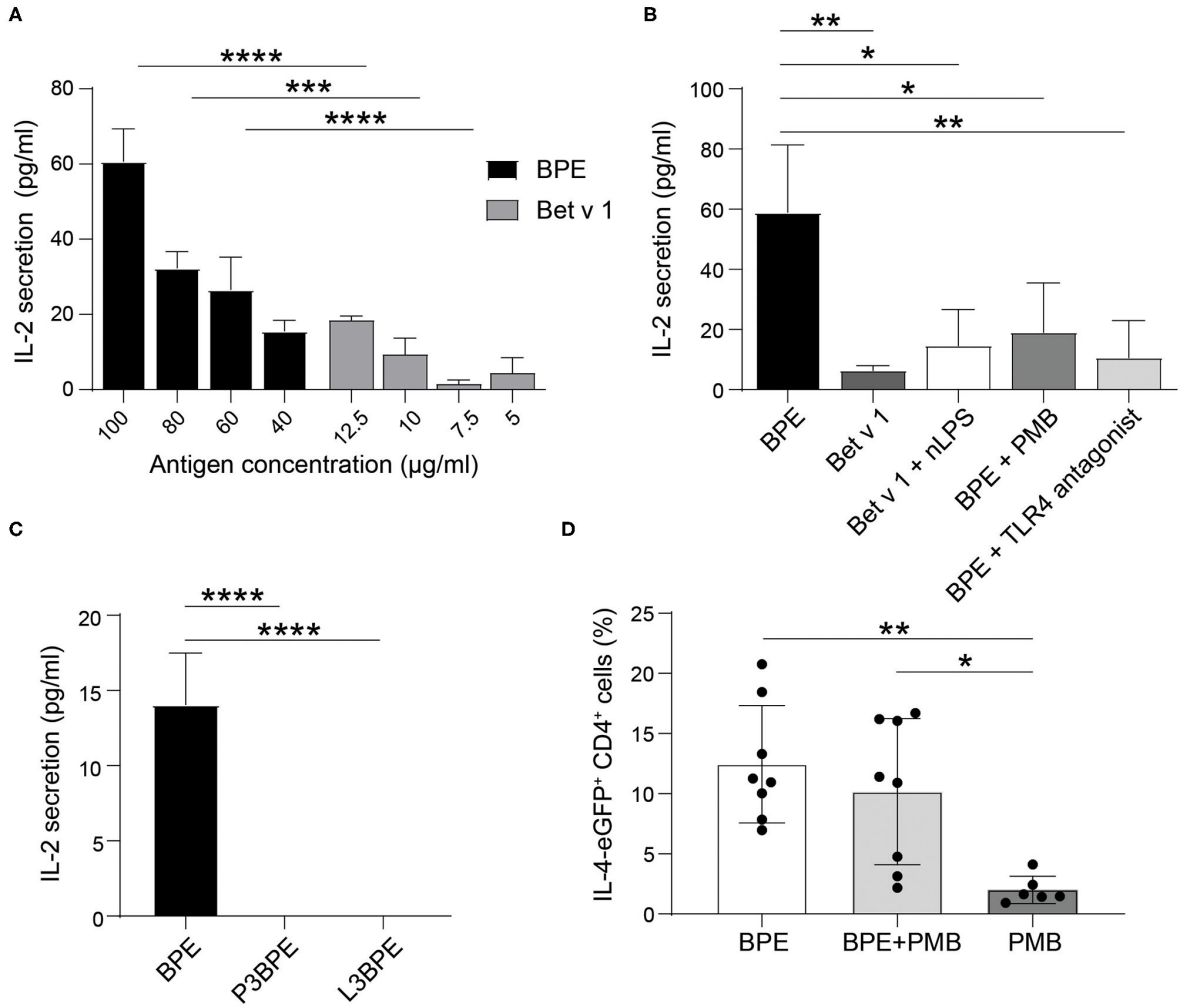
BPE-induced TLR4-dependent DC activation contributes to the T cell response *in vitro* and *in vivo*. The level of secreted IL-2 was analyzed in the supernatant of a 2-day co-culture of differentially primed-BMDCs and hybridoma T cells. BMDCs were stimulated with different concentrations of BPE (100, 80, 60, 40 μg/ml) or with the corresponding Bet v 1 concentration (12.5, 10, 7.5, 5 μg/ml, respectively) **(A)**. BMDCs were stimulated with either 80 μg/ml BPE or BPE pre-incubated for 1 h with 5 μg/ml PMB, or BMDCs were pre-treated for 1 h with 10.4 μg/ml TLR4 antagonist prior to BPE stimulation. As comparison, cells were stimulated with the corresponding nLPS (10.4 ng/ml) plus Bet v 1 **(B)**. BMDCs were stimulated with BPE or the protein- (P3) and lipid-free (L3) fractions thereof **(C)**. The control conditions, including untreated BMDCs and wells containing only T cells, did not induce any detectable signal, therefore, were set to zero. Statistical analysis was performed using a Two-Way ANOVA with a Sidak's multiple comparisons test comparing BPE and the corresponding Bet v 1 conditions **(A)** and a One-Way ANOVA with Dunnett's multiple comparisons test to the BPE reference **(B,C)**. IL-4 reporter mice (4get) were immunized with either 65 μg BPE (*n* = 8) or BPE with 3.5 μg PMB (*n* = 8) or PMB alone (*n* = 6) as negative control **(D)**. Data represent the frequency of IL-4-expressing CD4+ T cells found in the inguinal skin-draining lymph nodes. Statistics were calculated with a One-Way ANOVA with Sidak's multiple comparisons test.

We first conducted *in vitro* co-culture experiments with BPE-primed BMDCs (Supplementary Figure 7) and Bet v 1-specific hybridoma T cells. BMDCs stimulated with BPE induced a dose-dependent T cell activation, represented by the increased secretion of IL-2 into the culture supernatants (Figure 6A). This T cell activation was significantly stronger than that induced by BMDCs stimulated with the corresponding Bet v 1 concentration (12.5% of the total protein content in BPE), determined by sandwich ELISA (Supplementary Figure 8A). For example, BMDCs stimulated with 100 μg/ml BPE induced a secretion of 60 pg/ml IL-2, whereas the respective Bet v 1 (12.5 μg/ml) caused a 3-fold lower secretion of IL-2 of about 20 pg/ml. The stimulation of BMDCs with a combination of Bet v 1 and nLPS still induced significantly less IL-2 secretion compared to BMDCs stimulated with the corresponding BPE concentration (Figure 6B), suggesting that nLPS is not sufficient to reproduce the effect of BPE on T cell proliferation. To analyze how the DC activation via TLR4 influences the T cell response, BMDCs were either stimulated with BPE pre-incubated with 5 μg/ml PMB (ratio 1:20 PMB: BPE) or pre-treated with 10.4 μg/ml TLR4 antagonist (ratio 1:1,000 nLPS: TLR4 antagonist) before the T cells were added. The concentrations of PMB and TLR4 antagonist were chosen based on the BMDC activation assays, for which a complete inhibition of DC activation was observed (Figures 2C, 4A, respectively). The stimulation of BMDCs with BPE with both, PMB and TLR4 antagonist, prevented the secretion of IL-2 in the co-culture supernatants compared to BMDCs stimulated with BPE; IL-2 levels decreased from 60 to <20 pg/ml (Figure 6B). By examining the impact of the protein or lipid removal of BPE on the T cell response, we observed that both, P3- and L3BPE, completely lost their capacity to induce T cell activation in the co-culture system (Figure 6C). These data suggest that the protein digestion by the proteinase K was efficient, since the remaining peptides in BPE did not trigger IL-2 secretion by the Bet v 1-specific T cells. On the other hand, it suggests that BPE-derived lipids are necessary to stimulate BMDCs in order to elicit the following T cell response. Medium-treated BMDCs did not induce any detectable IL-2 signal. The IL-2 measured in the culture supernatant of the co-culture was unlikely derived from the stimulated BMDCs as the cytokine was not significantly secreted in response to 60 μg/ml BPE (Supplementary Figure 8B).

The intradermal immunization of IL-4 reporter mice with BPE caused a robust Th2 polarization as characterized by the increased percentage of IL-4/eGFP-expressing CD4+ T cells, reaching 7–20.7% (*n* = 8, average: 12.4%), in the draining lymph nodes (Figure 6D). In contrast, in mice immunized with BPE pre-incubated with the antibiotic PMB this effect was reduced by 2.2% in average (*n* = 8, 2.2–16.7%, average: 10.2%), and not significant. The control, PMB alone hardly induced Th2 polarization (*n* = 6, average: 2% IL-4/eGFP+ T cells).

## Discussion

In allergic sensitization, innate immune responses toward pollen sources are essential for the activation and priming of DCs that further promote the development of Th2 immune responses (66). Which compounds of the BP matrix are crucial for the initiation of this process are still unclear. Within this study, we shed light on the molecular mechanisms by which BP interacts with DCs and how this affects the initiation of the subsequent adaptive immune response. We demonstrated that (i) TLR4 is a major signaling pathway involved in the maturation of murine as well as human DCs by BPE *in vitro* and, given the presence of a BP-inhabiting microbiome, (ii) we investigated the extent of the contribution of nLPS. By decreasing the complexity of the BPE composition via fractionation, (iii) we showed that lipids rather than proteins account for the TLR4-mediated immunostimulatory activity of BPE. Finally, (iv) we exposed the contribution of the TLR4-mediated DC activation induced by BPE in activation of an effector T cell response *in vitro* and in *in vivo* Th2 polarization.

Numerous studies demonstrated the contribution of TLR4 in innate inflammatory responses that promote allergic airway sensitization (25, 67, 68). For instance, in an experimental allergic asthma model, an association between TLR4 and allergen-induced inflammation was found, as mice lacking a functional TLR4 pathway developed a reduced Th2 immune response with lower circulating ovalbumin-specific IgE levels in comparison to WT mice. Moreover, TLR4-KO DCs were less efficient in inducing inflammatory responses in the recipient after adoptive transfer compared to WT DCs (69). Alternatively, a mechanism by which IL-4 can bypass innate immune signals to induce Th2 polarization was described (70). However, it should be mentioned that the origin of initial IL-4 in the process of allergic sensitization remains uncertain. Since these findings were mostly performed with single antigens, such as ovalbumin and/or LPS as stimulus, the role of TLR4 upon activation by natural sources, such as allergenic pollen, is poorly understood. Therefore, comprehensive *in vivo* studies analyzing allergen-specific Th2 responses in models reproducing physiological exposures are of high relevance. Only few studies focused on the role of TLR4 in the context of pollen-induced allergic sensitization, which is also likely to differ between pollen species (28). In an allergic asthma model, BPE-induced airway inflammation was demonstrated to be dependent on TLR4, influencing immune cell infiltration and the local production of IL-4 and IL-10 cytokines, without affecting airway hyperresponsiveness (AHR) (71). This implies that TLR4 contributes to the priming of the early innate inflammatory response, further driving allergic airway sensitization induced by BPE. TLR4-KO mice failed to get sensitized to BPE as no BPE-specific IgE was detectable in the serum upon BPE immunization, implying and supporting our hypothesis that TLR4 contributes to the initiation of BP-induced allergic sensitization (42). In this respect, IgE class-switching was reported to be mediated by the TLR4 pathway, as for example Myd88-deficient B cells stimulated with LPS and IL-4 secreted reduced IgE levels compared to wild-type cells (72, 73). However, which innate immune cells are specifically modulated via TLR4 *in vivo* as well as which BPE-derived compounds contribute to the innate immune inflammation still need to be identified.

The herein described immunostimulatory activity of BPE induced an enhanced surface expression of co-stimulatory molecules on murine and human DCs. In human moDCs, the allergic status of the donors did not have an influence on their activation profile, which is in line with our former study showing no significant differences between atopic and non-atopic donors (29). In murine BMDCs, also the Th2-associated markers Jagged-1 and OX40L were augmented. The induction of Jagged-1 and -2 was shown in a previous study (74). Interestingly, OX40L is usually known to be indirectly up-regulated by epithelial cell-secreted TSLP (75). The observed stronger BMDC activation induced by nLPS compared to BPE could be due to the presence of GM-CSF needed for the generation of these cells, which results in high TLR4 expression levels, thus is influencing the sensitivity to LPS (76). Nevertheless, BMDCs from TLR4-KO mice were very useful to further confirm the TLR4-dependency in BPE-mediated DC activation. In agreement with our findings, it has been previously described that DCs lacking TLR4 are less efficiently activated and have an altered cytokine production when stimulated with BP (28). The down-regulation of the TLR4 surface expression observed upon BPE treatment could reflect a feedback loop mechanism to prevent prolonged signaling (35, 77, 78). In this respect, TLR4 activation was recently described to concomitantly trigger its own endocytosis in which CD14 plays a critical role (79). TLRs can also regulate the expression among each other, as they share common downstream pathways and cellular responses (80–83). This phenomenon requires attention in future studies, as TLR2 was also associated with allergic diseases (15, 16).

Anemophilous pollen, like BP, are natural vectors for bacteria (31, 32, 84), and thus contain LPS that can be sensed by TLR4-expressing cells in concentrations down to the picomolar range (85). Although numerous studies claimed an adjuvant role of LPS in promoting allergic airway inflammation in different models, including pollen allergy, the impact of the pollen-inhabiting microbiome and endotoxins on the development of allergen-specific Th2 responses and allergic airway sensitization is not fully understood (86). As stated by the hygiene hypothesis (87), the time and the duration of LPS exposure throughout the lifespan seems to be critical for its immunomodulatory effects, decreasing the risk of allergic sensitization in infancy or increasing the risk of respiratory symptoms to aeroallergens in sensitized adults (45, 88, 89). A major limitation in many studies addressing the role of LPS in the development of allergic sensitization relates to the use of LPS as a single entity and in an unphysiologically high dose instead of considering the natural amount of LPS present in the allergenic source. The latter depends on the pollen species, the microbial composition on the pollen as well as on environmental factors, including air pollution (32, 90). For studies working with pollen extracts, the amount of LPS may also vary according to the extraction method and/or the quantification method used. Several assays exist to detect LPS, however, due to its complex chemical structure, most of them have drawbacks (91). Here, we used two methods relying on distinct properties of the endotoxin, namely its capacity to signal via TLR4 using HEK reporter cells and to activate the factor C protein in the rFC assay. The second is an improved and more ethical variant of the LAL (Limulus amebocyte lysate) assay (92), bypassing potential false-positive signals due to interferences by glycans (1,3-b-D-glucan from molds and pollen) or proteases (93). The endotoxin levels in various types of pollen grains and pollen extracts including Japanese cedar, Japanese cypress, birch, ragweed, and grass, measured with the LAL assay, ranged from 23 to 1,100 pg per mg of pollen grains, the lowest in BP (28). This inter-species variation was associated with different activation capacities and cytokine responses in murine and human DCs *in vitro*. Recently, the endotoxin content was also evaluated in mugwort (*Artemisia vulgaris*) pollen and 40 other pollen types using the rFC assay and amounts below 20 EU/mg of pollen were found (57). This is in agreement with our LPS quantification in BPE, which was ~15 EU/mg BP. In this way, the endotoxin level determined in our BPE is representative compared to previous studies. The study reported that LPS is a crucial factor for the development of allergic lung inflammation in a murine model of mugwort pollen allergy (57). The authors observed higher levels of specific IgG_1_ in mice after intranasal instillation with an *Artemisia* pollen extract that contained a high level of LPS compared to an extract low in LPS. Specific IgE antibodies were not detected, hence, the results are in support, but not proof of a successful allergic sensitization.

Our results, showing an efficient inhibition of the TLR4 signaling by the antibiotic PMB as well as of the activation of murine and human DCs induced by both BPE and LPS, indicate a possible contribution of the endotoxin as immune stimulator in BPE. Candidate Gram-negative bacteria responsible for LPS contaminations of BPE are bacteria of the Caulobacterales, Enterobacteriales, Pseudomonadales, Sphingomonadales and Xanthomonadales order (31). Immobilized PMB is commonly used to remove contaminating endotoxin from blood in clinical medicine (94, 95), therefore, this method could be implemented to remove TLR4 agonists, especially LPS, from extracts used for allergen-specific immunotherapy in order to reduce an eventual risk of pyrogenicity and associated side-effects. However, because of its cationic property (96), PMB can bind other negatively charged molecules, including the protein kinase C, calmodulin and the house dust mite allergen, Der p 7 (97–99). Hence, the neutralization of other components than LPS in BPE by PMB cannot be excluded. Furthermore, we found that nLPS could only partially reproduce the intensity of activation induced by BPE in human moDCs, thus, the TLR4-mediated immunostimulatory activity possibly involves the BP microbiome-derived LPS but also implies the presence of additional TLR4 adjuvant(s) in BPE. In this respect, we recently showed that nLPS does not fully mimic the activation patterns induced by BPE in murine BMDCs as well as in human moDCs *in vitro* (29). Interestingly, although the major BP allergen Bet v 1 was found to bind various immunomodulatory lipidic ligands within its hydrophobic pocket modulating its allergenic property, no interactions between LPS and Bet v 1 could be measured (60). Besides, the TLR4-dependent neutrophilic airway inflammation induced by ragweed pollen extract could not be explained by the effect of LPS alone as the inflammatory response occurred independently of CD14, an essential coreceptor for LPS recognition (18).

Many different TLR4 ligands, either endogenous plant-derived or of exogenous nature, are described. These compounds exhibit a striking chemical diversity, comprising proteins (100), glycosaminoglycans (101), bivalent ions (102) and lipids (48, 93). In our study, BPE-derived proteins appeared to be dispensable for inducing TLR4 signaling and activating DCs. Instead, the contribution of lipids solubilized in our aqueous BPE appeared to be important. Either intrinsic or extrinsic (deriving from hosted bacteria) to the pollen matrix, pollen-associated lipids are recognized to display immunomodulatory properties such as attracting various immune cells, including DCs, and influencing allergic inflammation (103–107). Lipidomic profiling of over 20 different pollen types was conducted and led to the identification of about hundred different lipid classes, reflecting the high diversity of pollen lipids (108). Very recently, the lipid composition was analyzed in BP using different extraction methods investigating the modifications induced by atmospheric pollutants suggested to aggravate allergic sensitization (109). For example, plant-derived saturated fatty acids, such as palmitic acid, were found to signal via TLR4 (110–113), however, their direct interaction with the receptor needs clarification (114). Interestingly, the loss of TLR4 activity in L3BPE was not attributable to LPS as the endotoxin was not removed from BPE by the Cleanascite^TM^ reagent, further supporting the involvement of other TLR4 agonists within BPE. The Cleanascite^TM^ reagent was commonly used for the removal of lipids or lipoproteins from sera or culture supernatants (115–117), whereas no published data regarding the removal of LPS by the Cleanascite^TM^ reagent are available. Whether the partial loss of TLR4-dependent DC activation capacity observed for the L3 fraction compared to the original extract is due to only a partial contribution of BP lipids or to an incomplete removal of the lipids needs to be further investigated. In fact, the complex and heterogeneous composition of BPE renders lipid extraction difficult, therefore, the use of alternative lipid extraction methods (109), combined with comprehensive lipidomic analyses, for the specific targeting of LPS as well as for the identification of specific BP-derived TLR4 adjuvants are of utmost importance.

Our findings revealed the importance of BPE-induced TLR4-mediated DC activation in the promotion of the consecutive adaptive immune response and that this immunogenicity is hardly driven by the major allergen Bet v 1 but rather by additional key factor(s) within the BP matrix. Intriguingly, the addition of nLPS to the stimulation of DCs with Bet v 1 was not sufficient to mimic the BPE-induced T cell activation, suggesting that LPS plays a minor role in the priming of the adaptive immune response. The capacity of BPE-stimulated human moDCs to induce Th2 polarization *in vitro* co-culture models was demonstrated earlier (74, 105, 118). Moreover, we have recently demonstrated that Bet v 1 alone neither efficiently activates murine or human DCs *in vitro* nor induces Th2 polarization *in vivo*, whereas the complete BPE exerts these properties. Even upon depletion of Bet v 1, BPE maintains its Th2 polarizing capacity *in vivo* (29). This indicates that, although Bet v 1 is the major specific target of the adaptive immunity, the respective pollen context (here, the aqueous pollen extracts) is necessary to provide the full potential to initiate allergic sensitization. Moreover, by neutralizing the TLR4 ligands within BPE responsible for DC activation via PMB, we emphasize the importance of the TLR4-mediated DC activation induced by BPE for the induction of the adaptive T cell responses *in vitro* and *in vivo*. Although a minor tendency was observable *in vivo*, the Th2 polarization induced by BPE was not significantly decreased by PMB. Since the PMB dose was determined based on the capacity of PMB to completely inhibit DC activation *in vitro*, it is possible that the amount of PMB was not high enough in the mouse model. Future studies using *in vivo* sensitization models via the airways, with allergen-specific IgE production as read-out, will provide more insights into the key signals and mechanisms by which BP initiates allergic airway inflammation. Besides DCs, epithelial cells are also important players of the innate immunity expressing TLR4, thus, could also indirectly contribute to the activation of DCs and the overall inflammatory response during BP exposure. In this respect, the TLR4 signaling in airway structural and stromal cells was shown to be necessary for the establishment of inflammation and AHR in response to inhaled house dust mite (24, 119).

Taken together, we demonstrated that TLR4 is a major pathway for the activation of DCs by BPE, possibly involving TLR4 adjuvants of lipidic nature, and that this mechanism contributes to the stimulation of effector T cell responses. Considering the potential clinical aspect of our findings, the blockade of the TLR4 signaling pathway could offer new perspectives for prophylactic approaches, targeting the early immunological events of the disease pathogenesis. Preventive administration of TLR4 inhibitors prior to the BP flowering seasons could dampen innate immune activation and consequently, diminish disease progression via Th2-biased responses in susceptible individuals (71). Since TLR4 is involved in many antimicrobial defense mechanisms, TLR4 inhibitors could also increase the risk of infections and/or alter wound healing (120). Therefore, the identification of novel BP-derived TLR4 adjuvants open the possibility of specifically targeting the BP-induced TLR4 signaling without entirely blocking the protective function of this pathway.

## Data Availability Statement

The raw data supporting the conclusions of this article will be made available by the authors, without undue reservation.

## Ethics Statement

The studies involving human participants were reviewed and approved by technical university of Munich. The patients/participants provided their written informed consent to participate in this study. The animal study was reviewed and approved by Austrian Federal Ministry.

## Author Contributions

LP devised and performed experiments, wrote the manuscript, and created the figures. AK, FR, MT, MW, MK, and AB conducted experiments. CT-H and SG provided the human DC model. LA devised the experiments and led the study. All authors read the manuscript.

## Conflict of Interest

The authors declare that the research was conducted in the absence of any commercial or financial relationships that could be construed as a potential conflict of interest.

## Publisher's Note

All claims expressed in this article are solely those of the authors and do not necessarily represent those of their affiliated organizations, or those of the publisher, the editors and the reviewers. Any product that may be evaluated in this article, or claim that may be made by its manufacturer, is not guaranteed or endorsed by the publisher.

## Supplementary Material

Supplementary Figure 1 Interpolation using linear regression for the quantification of nLPS content in BPE using the mTLR4 HEK assay **(A)** or the rFC endotoxin detection assay **(B)**. BPE titration (based on the total protein concentration) and the LPS standard curve (LPS concentration) are represented in gray and black, respectively.

Supplementary Figure 2 Titration of soluble PMB in mTLR4 HEK reporter cells stimulated with 30 μg/ml BPE or 20 ng/ml LPS for 24 h **(A)**. Data are shown as smoothed trend lines for each treatment condition (smoothening function of 2nd order, 4 neighbors). Visualization of BPE samples from the PMB-pull-down assay using SDS-PAGE and silver staining **(B)**. From left to right: untreated BPE, the supernatant of BPE after PMB-pull-down (sn), the three washing steps following the pull-down procedure (W1, W2, W3) and the bead pellet resuspended in 10 μl reducing buffer. The upper band at 66 kDa corresponds to BSA, which was used for blocking unspecific binding to the beads. Murine TLR4 HEK assay comparing BPE **(C)** or LPS **(D)** before and after PMB-pull-down assays (left plots) or after pull-down assays with negative control (NC) beads (right plots). Serial dilutions of the samples facilitated the determination of differences in signal intensity and were used for calculating the AUC. Dotted line represents either medium- or PBS-treated control cells.

Supplementary Figure 3 Cytokine secretion profile of the BMDC activation assay investigating PMB inhibition shown in Figure 2. Statistics were calculated using a Two-Way ANOVA with a Dunnett's multiple comparisons test.

Supplementary Figure 4 Scheme showing the fractionation strategy of BPE, PBS, and nLPS for protein and lipid removal **(A)**. The protocol for protein degradation was repeated using negative control (NC) beads. Protein profile analyzed by SDS-PAGE and Coomassie Brilliant Blue staining before (BPE reference) and after proteinase K treatment for 30 min, 1, 2, or 24 h **(B)**. Proteins were completely degraded after 24 h, thus, chosen as experimental condition to generate the P3 fractions. The mTLR4 HEK assay was performed with PBS control fractions **(C)** and with BPE incubated with NC beads according to the proteinase K digestion protocol **(D)**. Dotted lines represent either medium- or PBS-treated control cells. Lipid removal was conducted for nLPS. The “lipid-free” (L3) fraction of nLPS was compared to the untreated nLPS in the mTLR4 HEK assay **(E)**. For statistical analysis, an unpaired Student's *t*-test of the AUC was used (ns, not significant). Cytokine secretion profile of BMDC activation assay investigating the BPE fractions **(F)**. A One-Way ANOVA with a Tukey's multiple comparisons test was used for statistical analysis.

Supplementary Figure 5 Cytokine secretion profile from the BMDC activation assay comparing BPE- and nLPS-stimulated WT with TLR4-KO C57/BL6 BMDCs **(A)**. Analysis of TLR4 surface expression in C57/BL6 BMDCs stimulated with either 20 μg/ml BPE or the corresponding nLPS concentration of 2.6 ng/ml over a period of 24 h **(B)**. The data are presented as percentage of viable TLR4+ CD11c+ cells. Dotted line represents the baseline level as determined by TLR4-KO BMDCs. Data are shown as smoothed trend line for each treatment condition (smoothening function of 2nd order, 4 neighbors). Statistics were calculated using a Two-Way ANOVA with either a Sidak's multiple comparisons test comparing WT and TLR4-KO **(A)** or a Tukey's multiple comparisons test **(B)**.

Supplementary Figure 6 Human moDCs were incubated with different concentrations of TLR4 antagonist prior to stimulation with 0.1 μg/ml LPS to determine the ratio of LPS:TLR4 antagonist necessary to achieve a complete inhibition (/ = no antagonist) **(A)**. An excess of 1,000-fold more TLR4 antagonist was used for the experiment in Figure 5B. The TLR4 antagonist was tested for unspecific moDC activation by stimulating the cells with 260 ng/ml TLR4 antagonist, which was the concentration used to inhibit 0.26 ng/ml nLPS in the experiment in Figure 5B **(B)**. Data represent two single, non-allergic **(A)** and allergic **(B)**, donors. Dotted line represents medium-treated control cells. The QQ plot verifies the normal distribution of the measured values for the TLR4 surface expression on human moDCs stimulated with BPE or nLPS presented in Figure 5C **(C)**.

Supplementary Figure 7 Gating strategy for the BMDC assays. Cells were gated in a classical FSC-A/SSC-A plot, then doublets and dead cells were removed in SSC-A/SSC-H and eF506/FSC-A, respectively **(A)**. Viable CD11c+ cells were gated by CD11c-PE/FSC-A based on unstained cells. Among living CD11c+ cells, inclusion gates were designed to avoid interferences derived from artifacts outside the analysis range, positive populations were analyzed in histograms for each marker (CD40, CD86, CD80, Jagged-1, OX40L, and FAS) **(B)**. Gating strategy for the TLR4 surface expression analysis was based on the unstained and TLR4-KO controls **(C)**.

Supplementary Figure 8 Interpolation using linear regression for the quantification of the Bet v 1 level in BPE via sandwich ELISA **(A)**. BPE titration (based on the total protein concentration) and the Bet v 1 standard curve are represented in green and blue, respectively. A concentration of 1 mg/ml BPE contained 0.125 mg/ml Bet v 1, representing about 12.5% of the total protein content in BPE. IL-2 levels measured in the culture supernatant of 60 μg/ml BPE-stimulated BMDCs seeded at a cell density of 2 × 10^5^ cells per well **(B)**. Data represent the pooled, normalized data of five individually performed BMDC activation assays, with the medium control set to 1.

Supplementary Table 1 Characteristics of the blood donors. Demographic information including the sex and the age, the allergic status, as well as the total IgE level (kU/L) and the sensitization profile of various allergic sources analyzed via ImmunoCAP tests are described for each donor.

## Abbreviations

AHR: airway hyperresponsiveness
AUC: area under the curve
BP: birch pollen
BPE: birch pollen extract
BMDC: bone marrow-derived dendritic cell
BSA: bovine serum albumin
BPE PMB sn: birch pollen extract supernatant of the PMB-pull-down
CD: cluster of differentiation
DC: dendritic cell
DPBS: Dulbecco's Phosphate-Buffered Saline
FACS: fluorescence-activated cell sorting
FSC: forward scatter
iFCS: heat-inactivated fetal calf serum
eGFP: enhanced green fluorescent protein
h: hour
HEK: human embryonic kidney
Ig: immunoglobulin
IL: interleukin
LAL: limulus amebocyte lysate
L3: lipid-free
LPS: lipopolysaccharide
min: minutes
MFI: mean fluorescence intensity
moDC: monocyte-derived dendritic cell
OD: optical density
OX40L: OX40 ligand
PRR: pathogen-recognition receptors
PBMC: peripheral blood mononuclear cell
PALM: pollen associated lipid mediator
PMB: polymyxin B
P3: protein-free
QQ: quantile-quantile
rFC: recombinant factor C
RT: room temperature
SD: standard deviation
SDS-PAGE: sodium dodecyl sulfate-polyacrylamide gel electrophoresis
SPF: specific pathogen free
SSC: side scatter
Th2: T helper type 2
TLR: toll-like receptor
TLR4: toll-like receptor 4
TLR4-KO: toll-like receptor 4-knock-out
WT: wild-type.
