# Supplementary figures and images for: Birch Pollen Induces Toll-Like Receptor 4-Dependent Dendritic Cell Activation Favoring T Cell Responses
Source: Front Allergy. 2021 Aug 12;2:680937. doi: 10.3389/falgy.2021.680937 (PMC8974861; doi:10.3389/falgy.2021.680937)

A

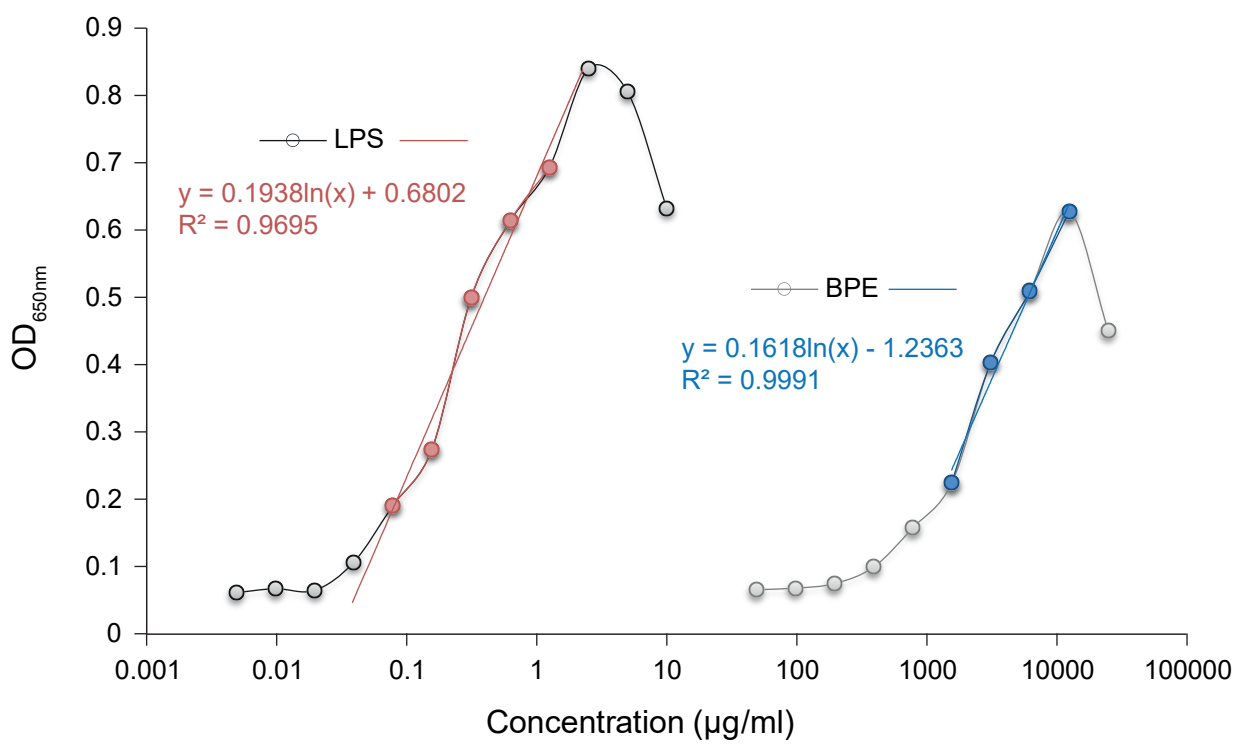

B

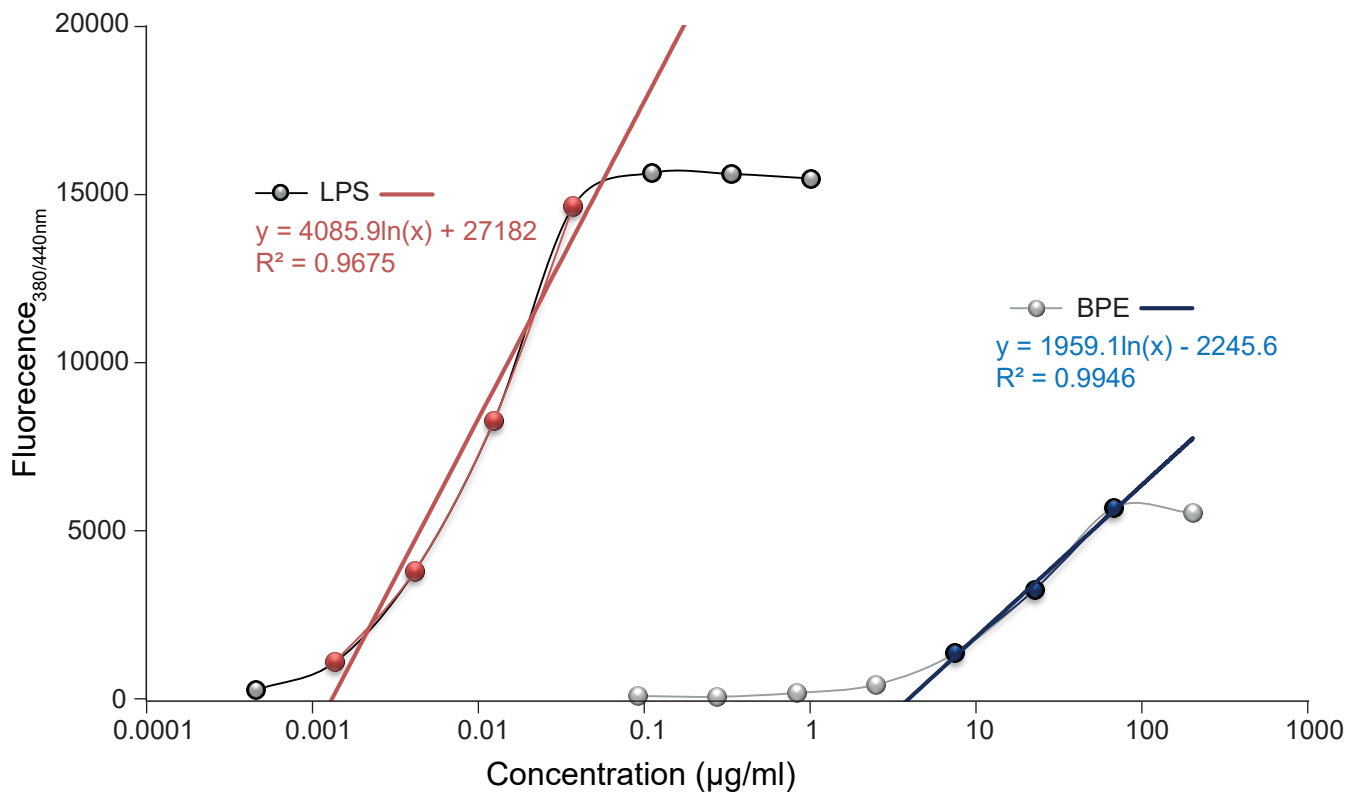

Supplement: Supplementary Figure 1 — Interpolation using linear regression for the quantification of nLPS content in BPE using the mTLR4 HEK assay (A) or the rFC endotoxin detection assay (B). BPE titration (based on the total protein concentration) and the LPS standard curve (LPS concentration) are represented in gray and black, respectively. [file Data_Sheet_1.PDF]

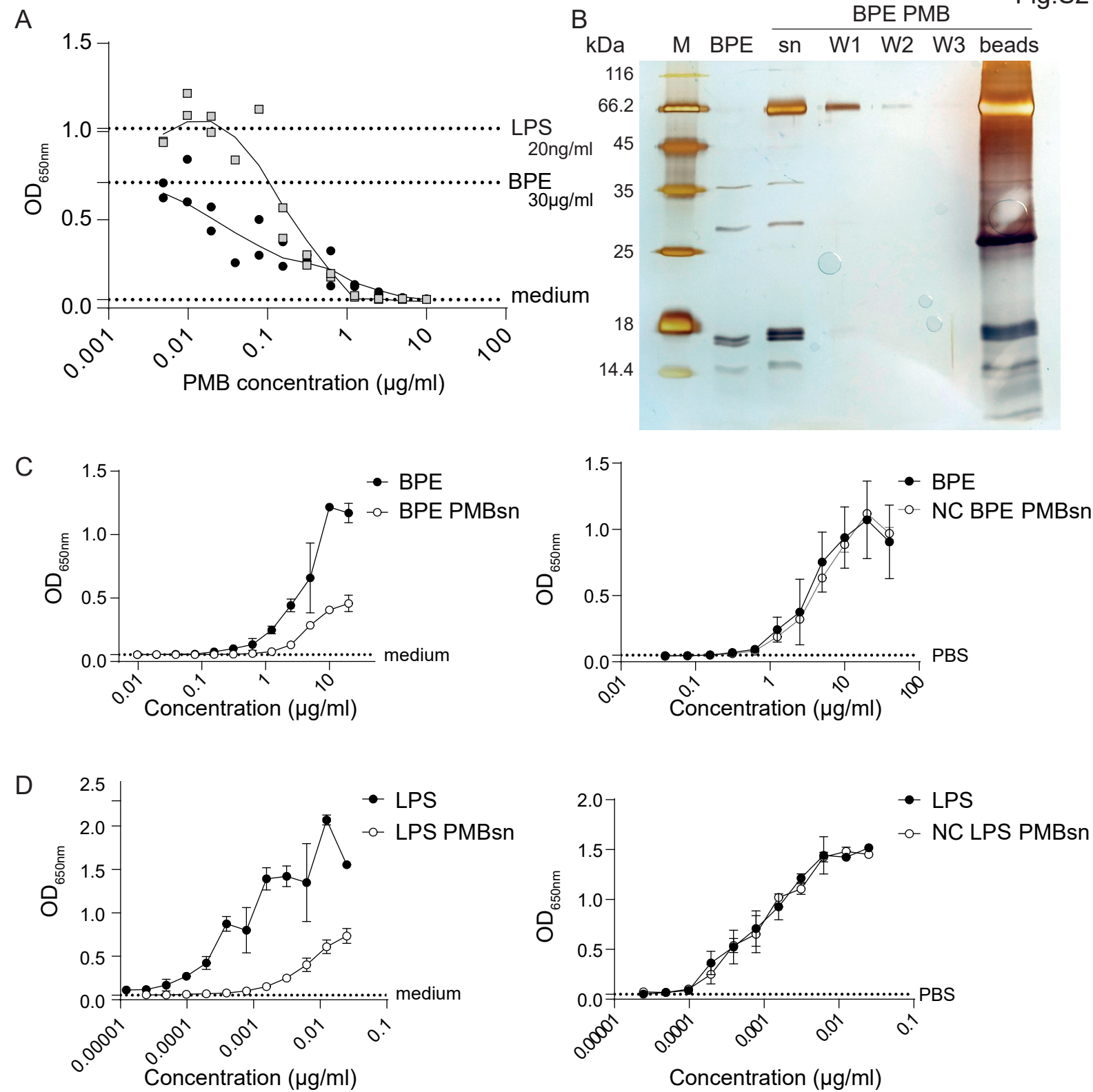

Supplement: Supplementary Figure 2 — Titration of soluble PMB in mTLR4 HEK reporter cells stimulated with 30 μg/ml BPE or 20 ng/ml LPS for 24 h (A). Data are shown as smoothed trend lines for each treatment condition (smoothening function of 2nd order, 4 neighbors). Visualization of BPE samples from the PMB-pull-down assay using SDS-PAGE and silver staining (B). From left to right: untreated BPE, the supernatant of BPE after PMB-pull-down (sn), the three washing steps following the pull-down procedure (W1, W2, W3) and the bead pellet resuspended in 10 μl reducing buffer. The upper band at 66 kDa corresponds to BSA, which was used for blocking unspecific binding to the beads. Murine TLR4 HEK assay comparing BPE (C) or LPS (D) before and after PMB-pull-down assays (left plots) or after pull-down assays with negative control (NC) beads (right plots). Serial dilutions of the samples facilitated the determination of differences in signal intensity and were used for calculating the AUC. Dotted line represents either medium- or PBS-treated control cells. [file Data_Sheet_2.PDF]

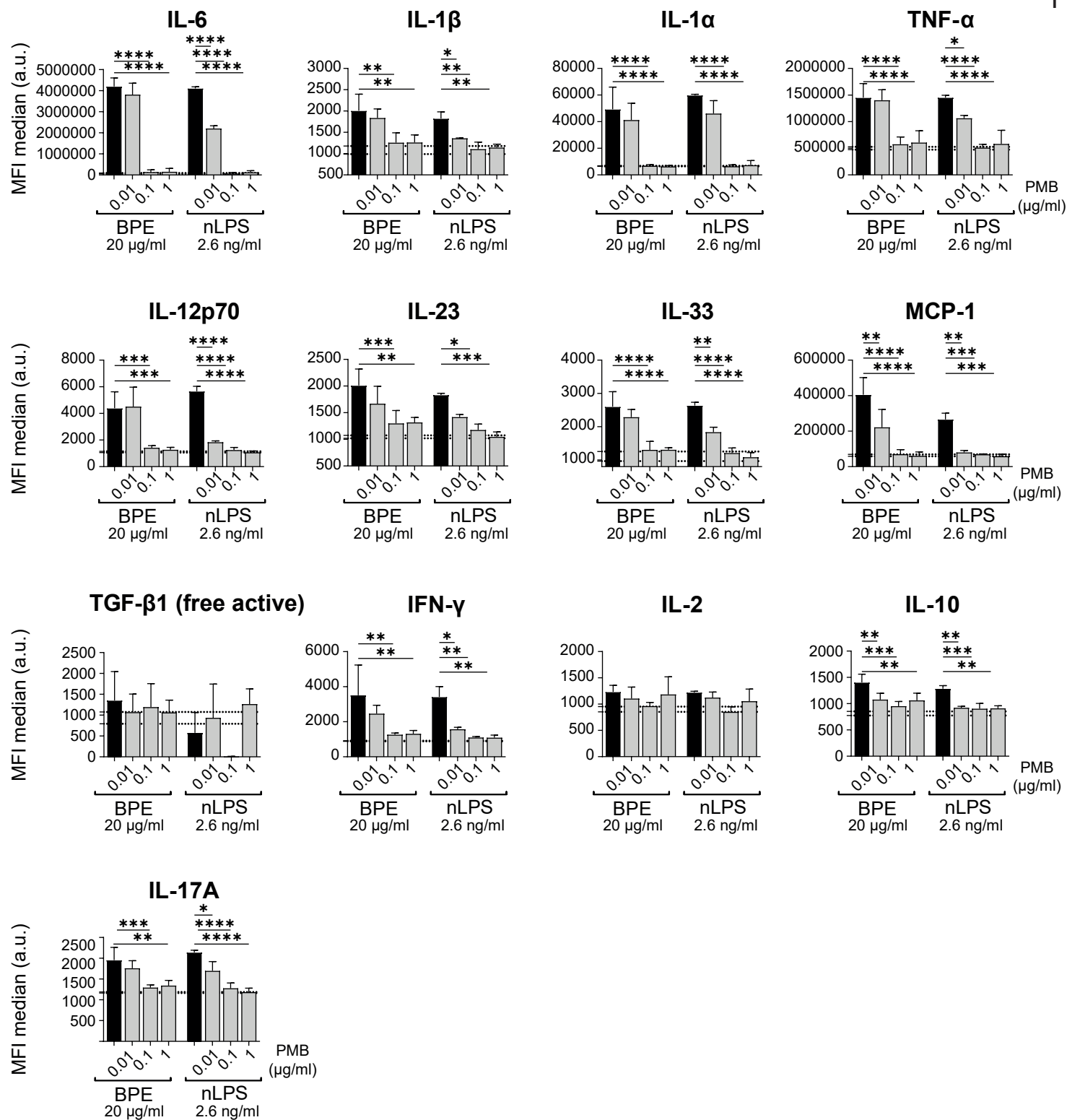

Supplement: Supplementary Figure 3 — Cytokine secretion profile of the BMDC activation assay investigating PMB inhibition shown in Figure 2. Statistics were calculated using a Two-Way ANOVA with a Dunnett's multiple comparisons test. [file Data_Sheet_3.PDF]

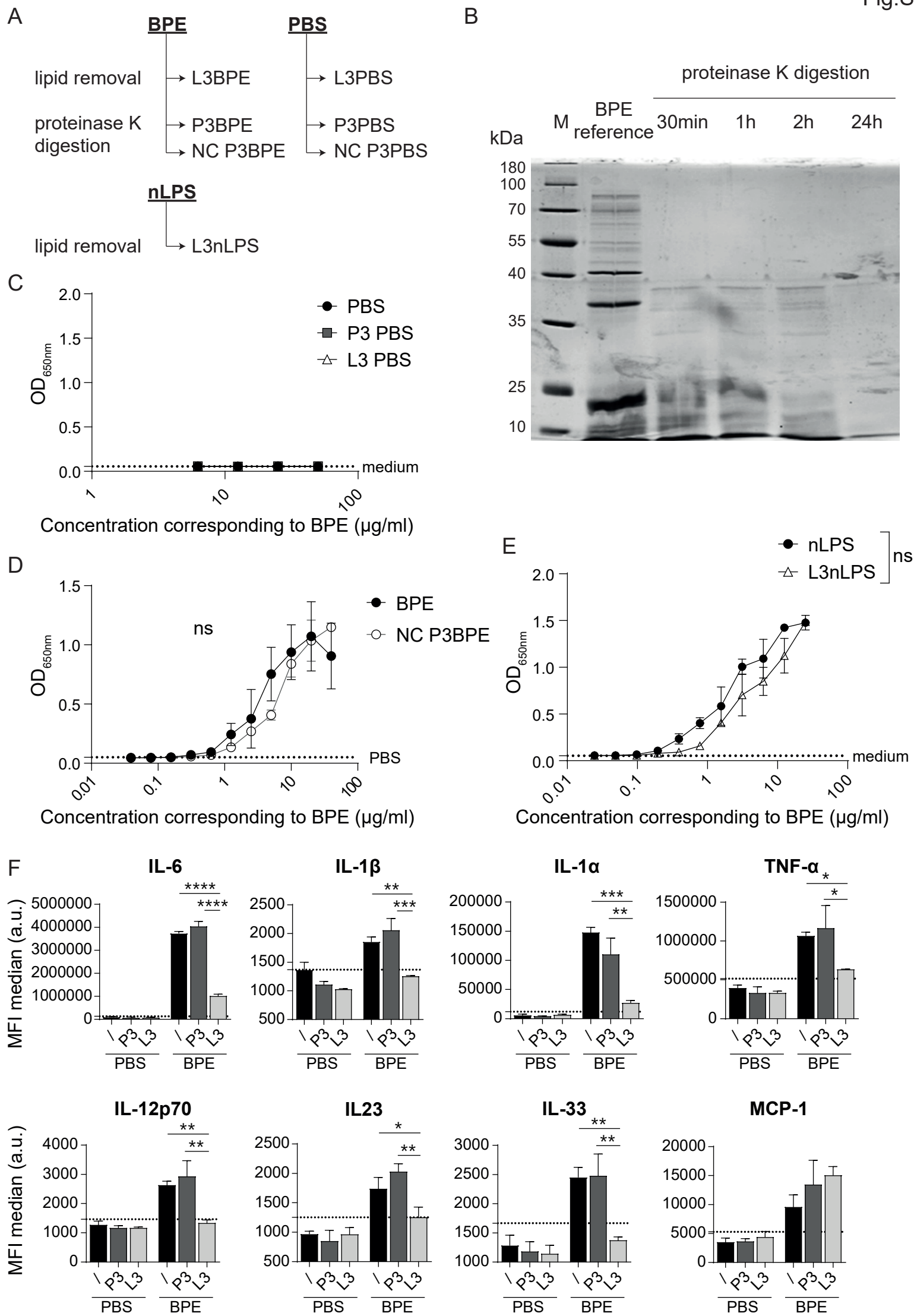

Supplement: Supplementary Figure 4 — Scheme showing the fractionation strategy of BPE, PBS, and nLPS for protein and lipid removal (A). The protocol for protein degradation was repeated using negative control (NC) beads. Protein profile analyzed by SDS-PAGE and Coomassie Brilliant Blue staining before (BPE reference) and after proteinase K treatment for 30 min, 1, 2, or 24 h (B). Proteins were completely degraded after 24 h, thus, chosen as experimental condition to generate the P3 fractions. The mTLR4 HEK assay was performed with PBS control fractions (C) and with BPE incubated with NC beads according to the proteinase K digestion protocol (D). Dotted lines represent either medium- or PBS-treated control cells. Lipid removal was conducted for nLPS. The “lipid-free” (L3) fraction of nLPS was compared to the untreated nLPS in the mTLR4 HEK assay (E). For statistical analysis, an unpaired Student's t-test of the AUC was used (ns, not significant). Cytokine secretion profile of BMDC activation assay investigating the BPE fractions (F). A One-Way ANOVA with a Tukey's multiple comparisons test was used for statistical analysis. [file Data_Sheet_4.PDF]

A

IL-6

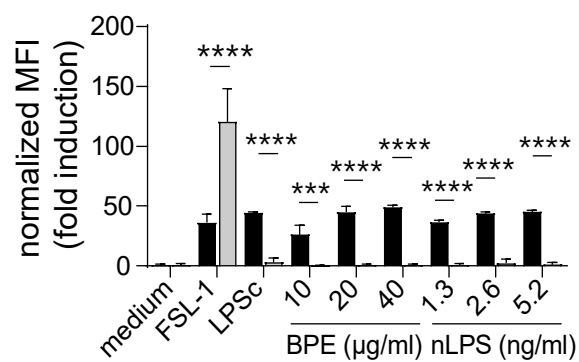IL-1 $\beta$ 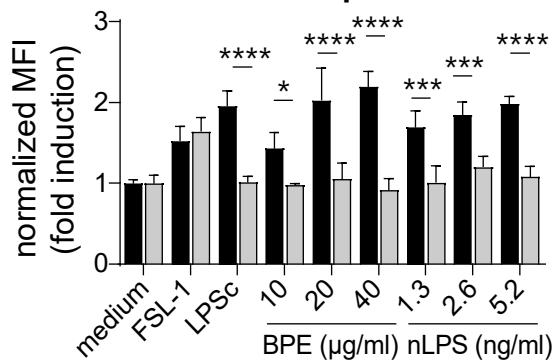IL-1 $\alpha$ 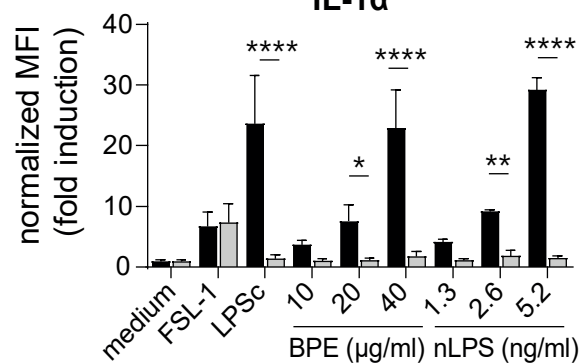TNF- $\alpha$ 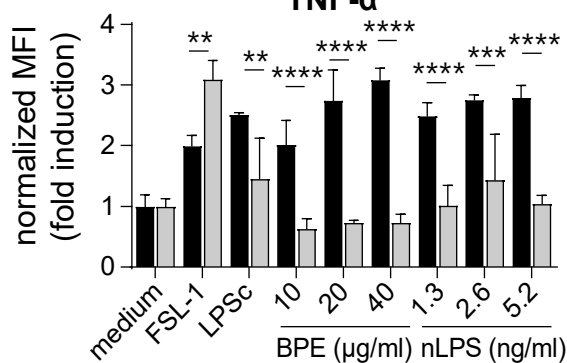

IL-33

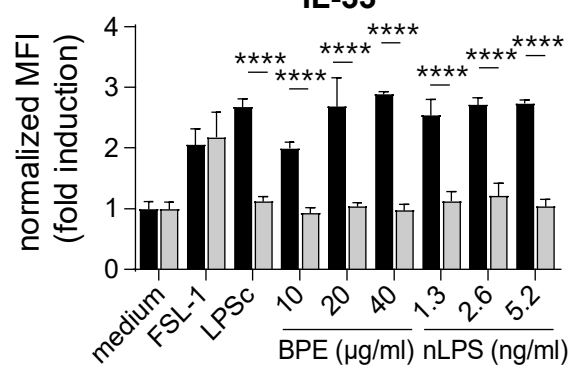

MCP-1

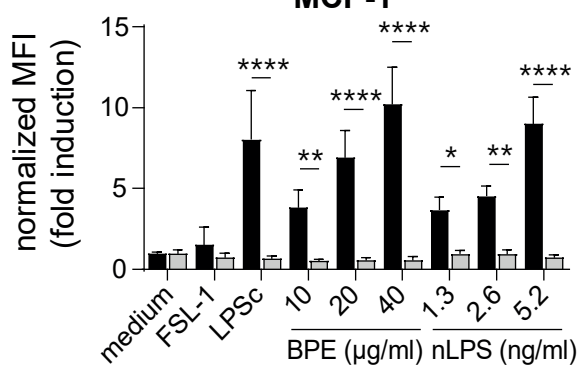

IL-12p70

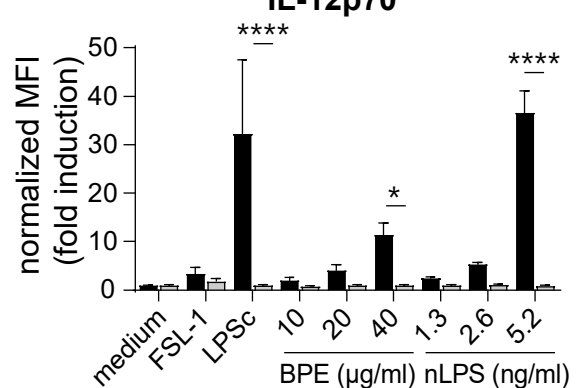

IL-23

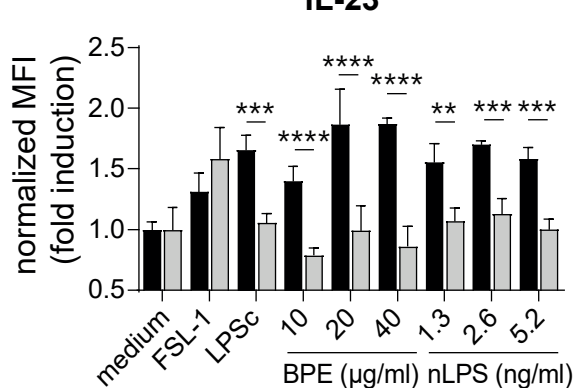

B

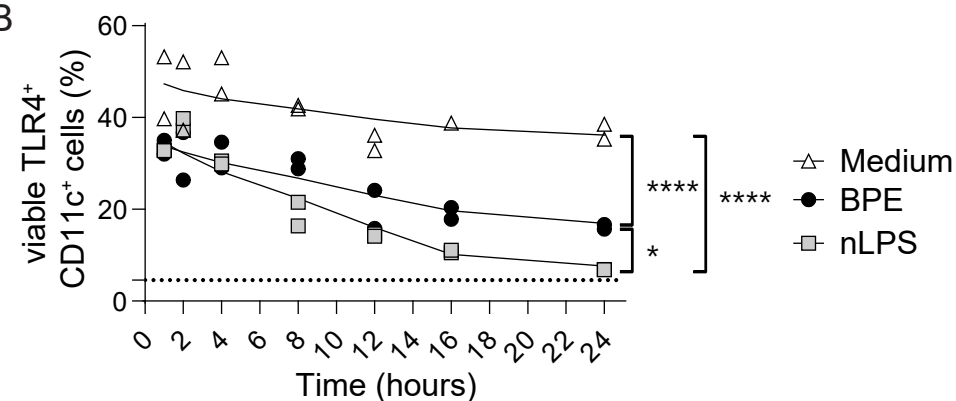

Supplement: Supplementary Figure 5 — Cytokine secretion profile from the BMDC activation assay comparing BPE- and nLPS-stimulated WT with TLR4-KO C57/BL6 BMDCs (A). Analysis of TLR4 surface expression in C57/BL6 BMDCs stimulated with either 20 μg/ml BPE or the corresponding nLPS concentration of 2.6 ng/ml over a period of 24 h (B). The data are presented as percentage of viable TLR4+ CD11c+ cells. Dotted line represents the baseline level as determined by TLR4-KO BMDCs. Data are shown as smoothed trend line for each treatment condition (smoothening function of 2nd order, 4 neighbors). Statistics were calculated using a Two-Way ANOVA with either a Sidak's multiple comparisons test comparing WT and TLR4-KO (A) or a Tukey's multiple comparisons test (B). [file Data_Sheet_5.PDF]

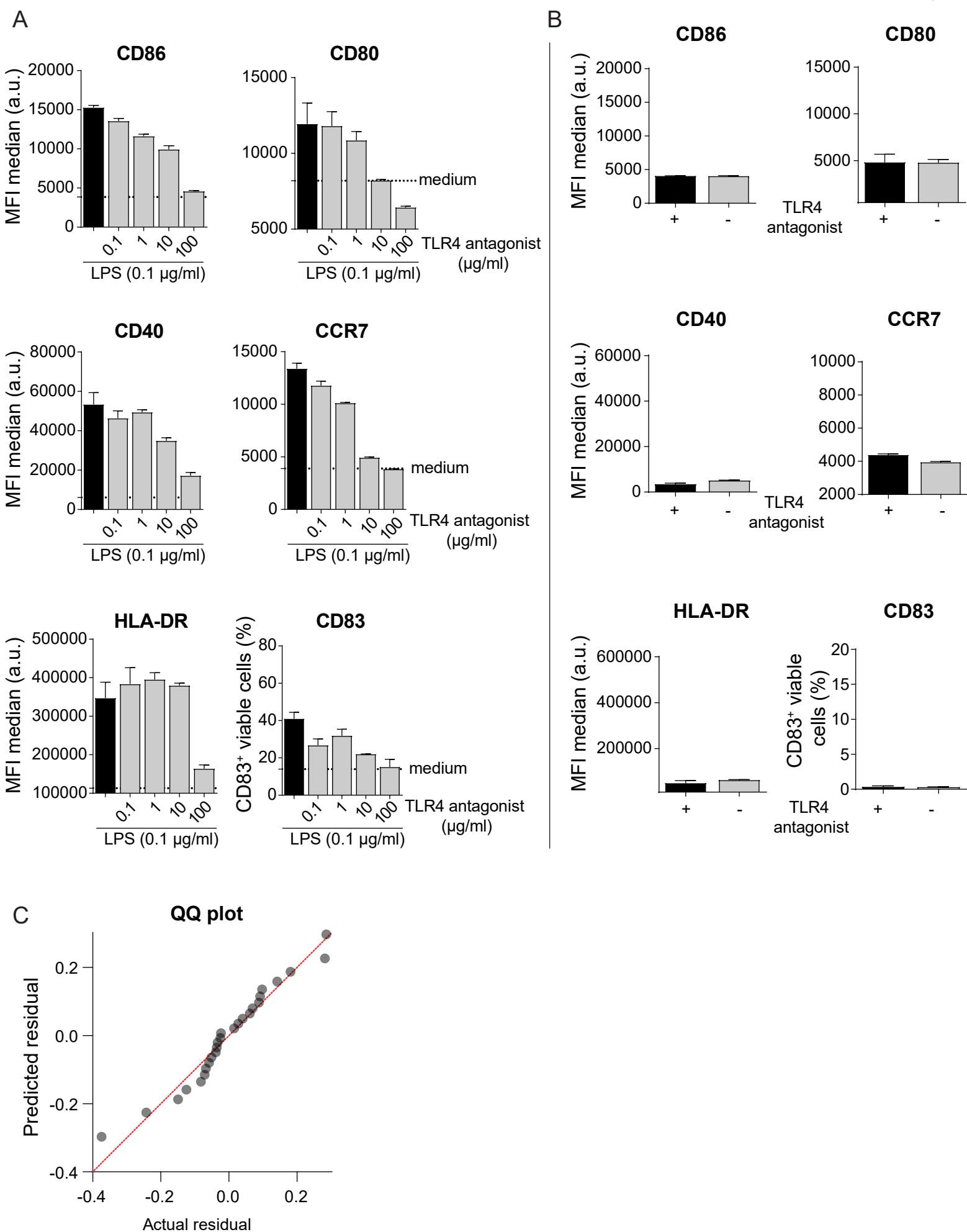

Supplement: Supplementary Figure 6 — Human moDCs were incubated with different concentrations of TLR4 antagonist prior to stimulation with 0.1 μg/ml LPS to determine the ratio of LPS:TLR4 antagonist necessary to achieve a complete inhibition (/ = no antagonist) (A). An excess of 1,000-fold more TLR4 antagonist was used for the experiment in Figure 5B. The TLR4 antagonist was tested for unspecific moDC activation by stimulating the cells with 260 ng/ml TLR4 antagonist, which was the concentration used to inhibit 0.26 ng/ml nLPS in the experiment in Figure 5B (B). Data represent two single, non-allergic (A) and allergic (B), donors. Dotted line represents medium-treated control cells. The QQ plot verifies the normal distribution of the measured values for the TLR4 surface expression on human moDCs stimulated with BPE or nLPS presented in Figure 5C (C). [file Data_Sheet_6.PDF]

A

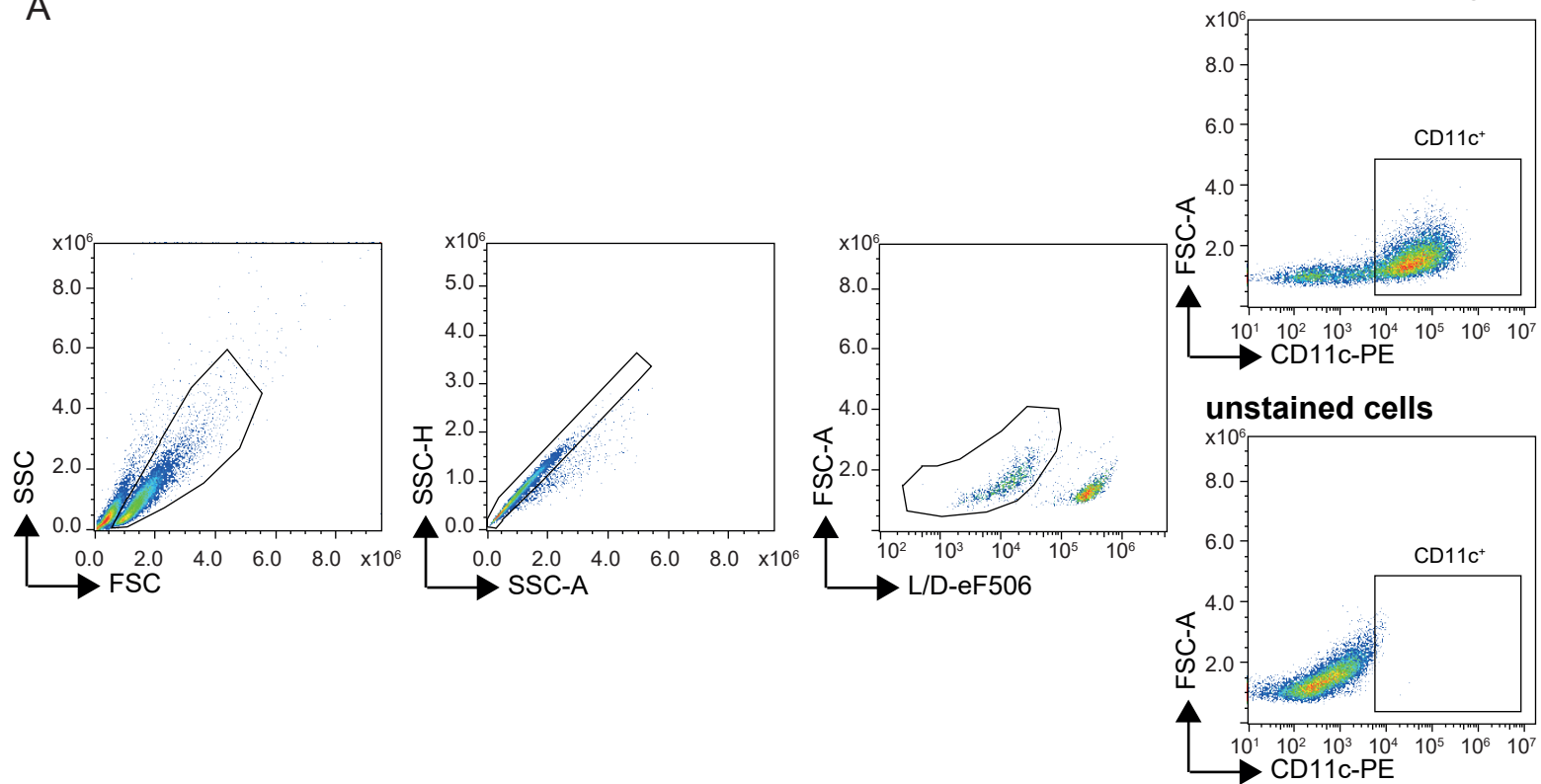

B

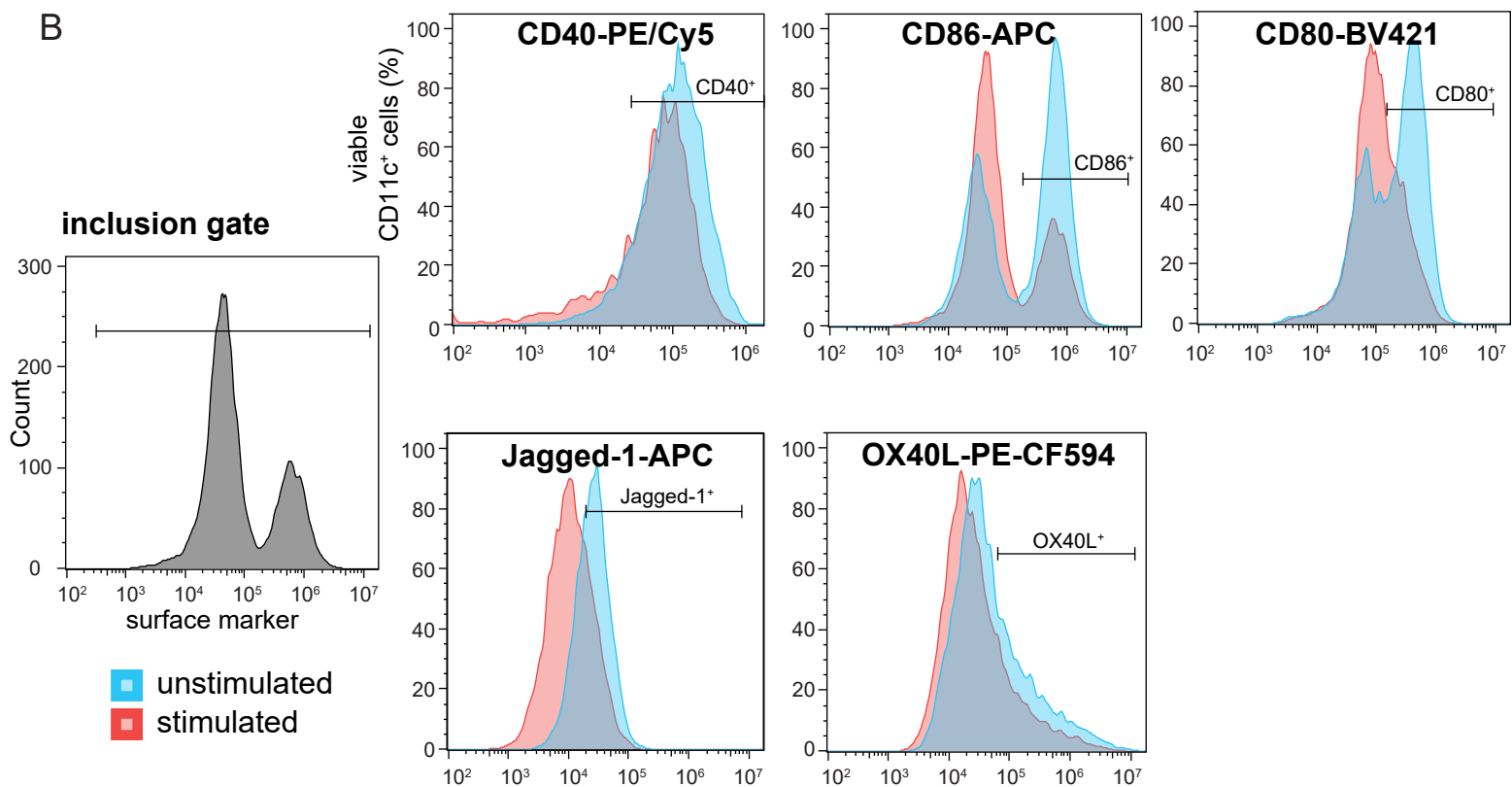

C

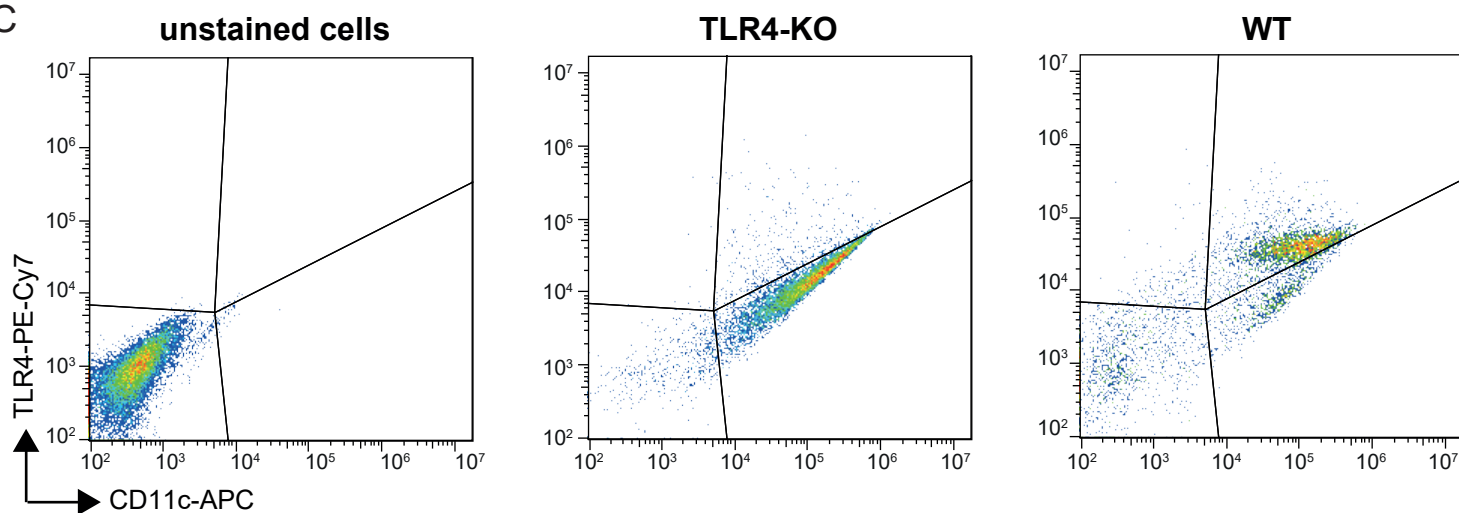

Supplement: Supplementary Figure 7 — Gating strategy for the BMDC assays. Cells were gated in a classical FSC-A/SSC-A plot, then doublets and dead cells were removed in SSC-A/SSC-H and eF506/FSC-A, respectively (A). Viable CD11c+ cells were gated by CD11c-PE/FSC-A based on unstained cells. Among living CD11c+ cells, inclusion gates were designed to avoid interferences derived from artifacts outside the analysis range, positive populations were analyzed in histograms for each marker (CD40, CD86, CD80, Jagged-1, OX40L, and FAS) (B). Gating strategy for the TLR4 surface expression analysis was based on the unstained and TLR4-KO controls (C). [file Data_Sheet_7.PDF]

A

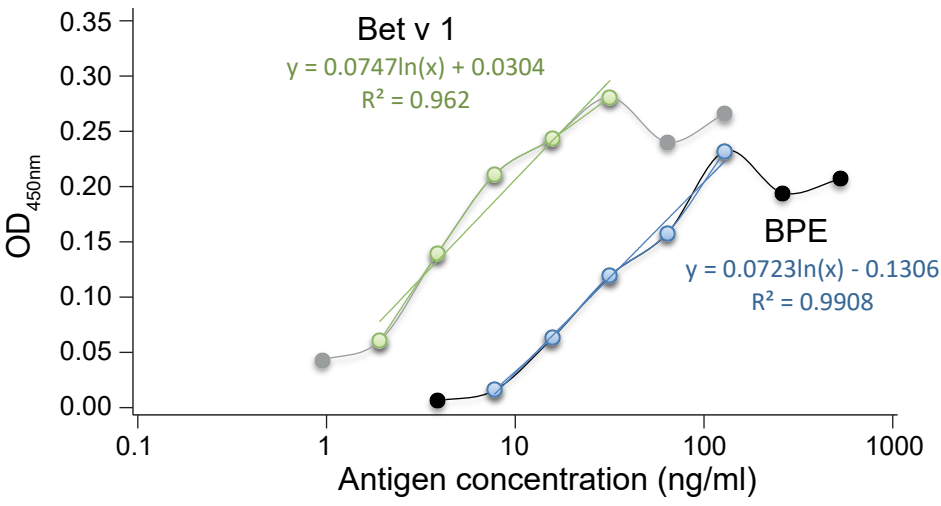

B

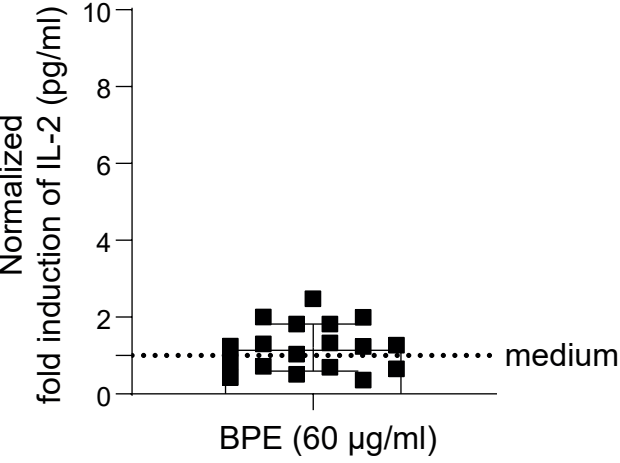

Supplement: Supplementary Figure 8 — Interpolation using linear regression for the quantification of the Bet v 1 level in BPE via sandwich ELISA (A). BPE titration (based on the total protein concentration) and the Bet v 1 standard curve are represented in green and blue, respectively. A concentration of 1 mg/ml BPE contained 0.125 mg/ml Bet v 1, representing about 12.5% of the total protein content in BPE. IL-2 levels measured in the culture supernatant of 60 μg/ml BPE-stimulated BMDCs seeded at a cell density of 2 × 10^5^ cells per well (B). Data represent the pooled, normalized data of five individually performed BMDC activation assays, with the medium control set to 1. [file Data_Sheet_8.PDF]
