# Supplementary material for: Birch Pollen Induces Toll-Like Receptor 4-Dependent Dendritic Cell Activation Favoring T Cell Responses
Source: Front Allergy. 2021 Aug 12;2:680937. doi: 10.3389/falgy.2021.680937 (PMC8974861; doi:10.3389/falgy.2021.680937)
Supplement: Supplementary Table 1 — Characteristics of the blood donors. Demographic information including the sex and the age, the allergic status, as well as the total IgE level (kU/L) and the sensitization profile of various allergic sources analyzed via ImmunoCAP tests are described for each donor. [file Table_1.DOCX]

|  |  |  |  |  |  | Specific IgE (kU/L) | | | | | | | | | | | | | |
| --- | --- | --- | --- | --- | --- | --- | --- | --- | --- | --- | --- | --- | --- | --- | --- | --- | --- | --- | --- |
| Figure | **Donor** | **Sex** | **Age** | **Allergic status** | **Total IgE (kU/L)** | **House dust mite** | **Cat** | **Dog** | **Timothy grass** | **Penicillium** | **Cladosporium** | **Alternaria** | **Alder** | **Birch** | **Hazel** | **Ash** | **Ambrosia** | **Mugwort** | **English plantain** |
| 5.A | 1 | F | 26 | NA | 18 | 0.1 | 0.1 | 0.1 | 0.1 | 0.1 | 0.1 | 0.1 | 0.1 | 0.1 | 0.1 | 0.1 | 0.1 | 0.1 | 0.1 |
| 5.B + S5 | 2 | F | 32 | NA | 2.79 | 0.01 | 0.01 | 0 | 0.01 | 0 | 0 | 0 | 0 | 0 | 0 | 0 | 0 | 0 | 0 |
| 5.C | 3 | F | 43 | A | 3.1 | **1.1*** | 0.1 | 0.1 | 0.1 | 0.1 | 0.1 | 0.1 | 0.1 | 0.1 | 0.1 | 0.1 | 0 | 0.1 | 0.1 |
| 5.C | 4 | F | 51 | A | 25 | **1.6*** | 0.1 | 0.1 | 0.1 | 0.1 | 0.1 | 0.1 | 0.1 | 0.1 | 0 | 0 | 0 | 0.1 | 0.1 |
| 5.C | 5 | F | 49 | A^◊^ | 420 | 37 | 25 | 6.1 | 9.7 | 0.4 | 1.4 | 8.5 | 45 | **58*** | 39 | 1.3 | 0 | 0.94 | 0.59 |
| 5.C | 6 | M | 43 | A | 15 | 0.1 | 0.1 | 0.1 | **2.4*** | 0.1 | 0 | 0.1 | 0.53 | 0.83 | 0.34 | 0.16 | 0.62 | 0.1 | 0.1 |
| S5 | 7 | F | 25 | A | 31 | 0.1 | 0.1 | 0.1 | **3.8*** | 0.1 | 0.1 | 0.1 | **2.9*** | **2.9*** | **1.1*** | 0.31 | 0 | 0.23 | 0.31 |

Table S1. Sensitization profile via ImmunoCap from blood donors for moDCs. All sampling were done out of the birch pollen season and none was under allergen immunotherapy treatment.

F, female; M, male; A, allergic; NA, non-allergic; *: expression of seasonal allergic symptoms; ^◊^: multi-sensitized donor (incl. birch)
